# Prevalence of group B *Streptococcus* colonisation in mother–newborn dyads in low-income and middle-income south Asian and African countries: a prospective, observational study

**DOI:** 10.1016/S2666-5247(24)00129-0

**Published:** 2024-10

**Authors:** Gaurav Kwatra, Alane Izu, Clare Cutland, Godwin Akaba, Musa Mohammed Ali, Zabed Ahmed, Manisha Madhai Beck, Hellen Cherono Barsosio, James A Berkley, Tolossa E Chaka, Anélsio Cossa, Sowmitra Chakraborty, Nisha Dhar, Phurb Dorji, Maksuda Islam, Adama Mamby Keita, Stella Mwakio, Salim Mwarumba, Nubwa Medugu, Helio Mucavele, Viviana Mabombo, Stephen Obaro, Betuel Sigaúque, Samba O Sow, Samir K Saha, Sridhar Santhanam, Ragunath Sharma, Eric A F Simoes, Rani Diana Sahni, Milagritos D Tapia, Balaji Veeraraghavan, Shabir A Madhi

**Affiliations:** a South Africa Medical Research Council Vaccines and Infectious Diseases Analytics Research Unit, Faculty of Health Science, University of the Witwatersrand, Johannesburg, South Africa; b African Leadership in Vaccinology Expertise, Faculty of Health Science, University of the Witwatersrand, Johannesburg, South Africa; c Wits Infectious Diseases and Oncology Research Institute, Faculty of Health Science, University of the Witwatersrand, Johannesburg, South Africa; d Division of Infectious Diseases, Department of Pediatrics, Cincinnati Children's Hospital Medical Center and University of Cincinnati College of Medicine, Cincinnati, OH, USA; e Department of Clinical Microbiology, Christian Medical College, Vellore, India; f Fetal Medicine Unit, Christian Medical College, Vellore, India; g Department of Neonatology, Christian Medical College, Vellore, India; h Department of Obstetrics and Gynaecology, The University of Abuja Teaching Hospital, Abuja, Nigeria; i Hawassa University College of Medicine and Health Sciences, School of Medical Laboratory Sciences, Hawassa, Ethiopia; j Child Health Research Foundation, Dhaka, Bangladesh; k Bangladesh Shishu Hospital and Institute, Dhaka, Bangladesh; l KEMRI/Wellcome Trust Research Programme, Kilifi, Kenya; m Department of Pediatrics and Child Health, Adama Hospital Medical College, Adama City, Ethiopia; n Centro de Investigação em Saúde da Manhiça (CISM), Manhica, Mozambique; o Jigme Dorji Wangchuck National Referral Hospital, Thimphu, Bhutan; p Le Centre pour le Développement des Vaccins du Mali (CVD-Mali), Bamako, Mali; q International Foundation Against Infectious Diseases in Nigeria, Abuja, Nigeria; r Department of Medical Microbiology and Immunology, Nile University of Nigeria, Abuja, Nigeria; s Department of Pediatrics, Children's Hospital Colorado, University of Colorado School of Medicine, Colorado School of Public Health, Aurora, CO, USA; t Center for Vaccine Development and Global Health, University of Maryland School of Medicine, Baltimore, MD, USA

## Abstract

**Background:**

Rectovaginal group B *Streptococcus* (GBS) colonisation in pregnant individuals at the time of labour is a major risk factor for invasive GBS disease by age 7 days (early-onset disease). We aimed to investigate the prevalence of rectovaginal GBS colonisation at the time of labour among pregnant women and vertical transmission to their newborns across selected low-income and middle-income African and south Asian countries.

**Methods:**

This prospective, observational study was undertaken at 11 maternity and obstetric care facilities based in Ethiopia, Kenya, Mozambique, Nigeria, Mali, South Africa, Bangladesh, India, and Bhutan. HIV-negative pregnant women aged 18–45 years who were in the early stages of labour and at least 37 weeks’ gestation were eligible for inclusion. Lower vaginal and rectal swabs and urine were collected from the women, and swabs of the umbilicus, outer ear, axillary fold, rectum, and throat were obtained from their newborns, for GBS culture. Standardised sampling and culture using direct plating and selective media broth for detection of GBS colonisation was undertaken at the sites. Serotyping of GBS isolates was done in South Africa. The primary outcome was the prevalence of rectovaginal GBS among pregnant women, analysed in participants with available data. This study is registered with the South African National Clinical Trials Register, number DOH-27–0418–4989.

**Findings:**

6922 pregnant women were enrolled from Jan 10, 2016, to Dec 11, 2018, of whom 6514 (94·1%; 759–892 per country) were included in the analysis; data from Bhutan were not included in the study due to issues with specimen collection and processing. Overall, the prevalence of maternal GBS colonisation was 24·1% (95% CI 23·1–25·2; 1572 of 6514); it was highest in Mali (41·1% [37·7–44·6]; 314 of 764) and lowest in Ethiopia (11·6% [9·5–14·1]; 88 of 759). The overall rate of vertical transmission of GBS from women with rectovaginal GBS colonisation was 72·3% (70·0–74·4; 1132 of 1566); it was highest in Mozambique (79·2% [73·3–84·2]; 168 of 212) and lowest in Bangladesh (55·8%, 47·5–63·8; 77 of 138). The five most common GBS colonising serotypes were Ia (37·3% [34·9–39·7]; 586 of 1572), V (28·5% [26·3–30·8]; 448 of 1572), III (25·1% [23·0–27·3]; 394 of 1572), II (9·2% [7·8–10·7]; 144 of 1572), and Ib (6·5% [5·4–7·8]; 102 of 1572). There was geographical variability in serotype proportion distribution; serotype VII was the third most common serotype in India (8·6% [5·3–13·7]; 15 of 174) and serotype VI was mainly identified in Bangladesh (5·8% [3·0–11·0]; eight of 138) and India (5·7% [3·2–10·3]; ten of 174).

**Interpretation:**

Our study reported a high prevalence of GBS colonisation in most settings, with some geographical variability even within African countries. Our findings suggest that serotypes not included in current multivalent capsular-polysaccharide GBS vaccines prevail in some regions, so vaccine efficacy and post-licensure effectiveness studies should assess the effect of vaccination on maternal GBS colonisation given the potential for replacement by non-vaccine serotypes.

**Funding:**

Bill & Melinda Gates Foundation.

## Introduction

Invasive bacterial disease and pneumonia contribute to approximately one-fifth of deaths in neonates globally, with more than 90% occurring in low-income and middle-income countries (LMICs).^1^ Group B *Streptococcus* (GBS) is a leading cause of invasive bacterial disease in infants younger than 90 days. Globally, in 2020, there were an estimated 394 000 cases of invasive GBS disease in infants younger than 90 days, including 90 000 deaths.^2^ Peripartum acquisition of GBS by the fetus or newborn from mothers with rectovaginal GBS colonisation is a major risk factor for developing invasive GBS disease during the first 7 days of life (early-onset disease).^3^ In the absence of intrapartum antibiotic prophylaxis in pregnant individuals who have been colonised with GBS between 32 and 36 weeks’ gestation, approximately 1–2% of their newborns are estimated to develop early-onset invasive GBS disease.^3^ Maternal rectovaginal colonisation by GBS might also contribute to illness during pregnancy and postpartum, and might contribute to at least 57 000 stillbirths and 3·5 million preterm births worldwide per year.^4^

**Research in context**

**Evidence before this study**

Peripartum acquisition of group B *Streptococcus* (GBS) by the fetus or newborn from mothers with rectovaginal GBS colonisation is the major risk factor for invasive GBS disease during the first 7 days of life (early-onset disease). A systematic review of all GBS colonisation studies published until Jan 31, 2017, reported that the prevalence of rectovaginal GBS colonisation in pregnant women ranged from 2·0% in Melanesia to 33·5% in the Caribbean region. Differences between studies in sample collection techniques, timing of sampling during pregnancy and in the newborn, and microbiological culture methods for GBS were identified as potential factors that could have contributed to the variability in prevalence of GBS colonisation and vertical transmission across the studies. We searched PubMed for reports published between Jan 1, 2016, and May 31, 2023, using the keywords (“Group B Streptococcus (MeSH term)” OR “Streptococcus agalactiae (MeSH term)” AND “Colonization” with no restrictions on language, and found 470 articles on GBS colonisation. We identified 33 observational studies reporting on rectovaginal GBS colonisation among pregnant women, which included 14 studies from Africa and two from south Asia. Of these studies, only five reported a GBS vertical transmission rate (range 7·6–63·3%) and none reported data on GBS bacteriuria. Maternal GBS colonisation ranged from 12·3% to 37% in Africa and from 14·8% to 14·9% in south Asia. Different culture methods, sample collection techniques, and timings of sampling during pregnancy were reported in these studies.

**Added value of this study**

Using standardised methods across all the sites, our study provides more robust data than previously existed, to enable head-to-head comparability of geographical differences in the prevalence of GBS colonisation in pregnant women and vertical transmission of GBS to their newborns. The overall prevalence of maternal GBS colonisation was 24·1% (95% CI 23·1–25·2) in our study: 25·8% in women from Africa and 19·0% in women from south Asia. Also, we identified geographical heterogeneity in serotype distribution of colonising isolates, particularly serotypes VII and VI, which were among the top five serotypes in south Asian countries. Our study also identified high rates of vertical transmission (55·8–79·2%) of GBS from colonised women to their newborns, with maternal GBS bacteriuria being a strong predictor of vertical transmission across all the sites.

**Implications of all the available evidence**

Standardised approaches to investigate maternal rectovaginal GBS colonisation and vertical transmission of GBS can provide robust data and enable more reliable intercountry comparisons of maternal GBS rectovaginal colonisation and rate of vertical transmission to newborns than has been possible previously. Based on the estimate that 1–2% of babies born to women with rectovaginal GBS colonisation develop invasive GBS disease in the absence of intrapartum antibiotic prophylaxis, newborns are likely to be at high risk of invasive GBS disease in the countries assessed in our study. The current hexavalent GBS polysaccharide–protein conjugate vaccine (GBS6) being evaluated in pregnant women includes six serotypes (Ia, Ib, II, III, IV, and V); these six serotypes accounted for 97·3% of all maternal colonising isolates in our study, ranging from 100% in Ethiopia and Kenya to 85·8% in India. Nevertheless, considering that serotypes not included in GBS6 prevail in some regions, vaccine efficacy and post-licensure effectiveness studies should assess the effect of vaccination on maternal GBS colonisation given the potential for replacement by non-vaccine serotypes.

A systematic review reported that the prevalence of rectovaginal GBS colonisation in pregnant women ranged from 2·0% in Melanesia to 33·5% in the Caribbean region.^5^ The reported rate of vertical transmission of GBS from pregnant women to their newborns ranged from 7·6% to 63·3%.^6^, ^7^, ^8^ Differences between studies in sample collection techniques, timing of sampling during pregnancy and in the newborn, and microbiological culture methods for GBS might have contributed to the great variability in prevalence of GBS colonisation and vertical transmission rates.^5^^,^^9^ Consequently, standardised methods are required to improve comparison of the prevalence of maternal GBS colonisation and vertical transmission to newborns in different settings. Improved understanding of the prevalence of GBS colonisation in pregnant individuals, serotype distribution, and dynamics of transmission from mothers to their newborns could also assist in clarifying the variability reported in the incidence of early-onset invasive GBS disease in different settings, which has been estimated to be 0·71 per 1000 livebirths in Africa and 0·32 per 1000 livebirths in Asia.^5^^,^^10^

The aim of this study was to investigate the prevalence of GBS rectovaginal colonisation and serotype distribution among pregnant women at the time of delivery in African and south Asian LMICs using standardised methods. Furthermore, we evaluated the prevalence of maternal GBS bacteriuria and vertical transmission of GBS to the newborns.

## Methods

### Study design and participants

We conducted a multicountry, prospective, observational study at 11 maternity and obstetric care facilities based in six African countries (Ethiopia [Adama], Kenya [Kilifi], Mozambique [Manhica], Nigeria [Gwagwalada], Mali [Bamako], and South Africa [Johannesburg]) and three south Asian countries (Bangladesh [Mirzapur], Bhutan [Thimphu], and India [Vellore]; appendix p 2).

Participants were screened for enrolment during the early stages of labour. All eligible pregnant women who presented at the facilities during the daytime, Monday to Friday, were approached for participation in the study, although the maximum number enrolled per day was determined at the individual site level. Participants were eligible if they were pregnant women aged 18–45 years who were evaluated to be at least 37 weeks’ gestation on the basis of the date of their last menstrual period and corroborated by physical examination, or ultrasound examination if available, and who were HIV-negative. Participants’ HIV status was obtained from their medical records if tested during the course of the current pregnancy; if it had not, they were tested through routine care services before enrolment in the study. Testing of women for HIV-1 was not undertaken in Bangladesh, as the population prevalence of maternal HIV-1 was less than 1%.^11^ Exclusion criteria were reported use of antibiotics in the 2 weeks before delivery, any acute illness, symptomatic vaginal discharge and a known or suspected condition in which vaginal examination and swab collection were contradicted, inability to obtain a blood sample, or having received a blood transfusion in the 30 days before delivery (appendix p 5).

The study was approved by the Human Research Ethics Committee (Medical) of the University of the Witwatersrand (150214), and site-specific ethics approval was obtained from ethics committees of all the participating sites’ institutions. Written informed consent was obtained from all participating mothers. The protocol is provided in the appendix (pp 29–67). This study is registered with the South African National Clinical Trials Register, number DOH-27–0418–4989.

### Procedures

Standardised methods for sample collection, bacterial culture, and serotyping were undertaken across all the sites (appendix p 5). Laboratory scientists and clinical staff from all the participating sites received training applicable to their responsibilities in accordance with the Wits Vaccines and Infectious Diseases Analytics Research Unit (Wits-VIDA) laboratory, Johannesburg (South Africa), and clinical standard operating procedures (appendix pp 3–4). All laboratory and clinical supplies including culture media required to collect and process specimens were supplied once every 2 weeks to the sites from Wits-VIDA. Separate lower vaginal and rectal swabs and urine samples were collected from the women during labour and processed for GBS culture. Separate skin surface swabs of the umbilicus, outer ear, and axillary fold, and rectal and throat swabs, were obtained from the baby immediately after birth and before cleansing or bathing. All the swabs were collected by trained clinical study staff and the urine samples were collected by the participants under the supervision of the study staff. Swabs and urine, which was tested for the presence of antimicrobials, were processed at onsite laboratories according to standard US Centers for Disease Control and Prevention guidelines^3^ (appendix p 6). Briefly, swabs were inoculated onto selective media (CHROMagar StrepB, Media Mage, Johannesburg, South Africa; a direct plating method) and into selective broth (Todd–Hewitt broth supplemented with colistin and nalidixic acid [LIM broth]; BD, Maryland, USA; catalogue number 296266). For Ethiopia and Mali, due to logistical issues associated with delivery of consumables and reagents to the sites, swab samples were stored in a storage medium containing skimmed milk, tryptone, glucose, and glycerin (STGG) and shipped to Wits-VIDA for GBS culturing. Urine samples from Mali and Ethiopia were frozen and shipped to Wits-VIDA for the purposes of GBS culture.

The GBS isolates from all sites were stored in STGG medium at –70°C and shipped to Wits-VIDA for serotyping. Serotyping was performed on all GBS isolates using a latex agglutination assay with specific antisera against types Ia, Ib, II, III, IV, V, VI, VII, VIII, and IX capsular polysaccharide antigens (Statens Serum Institut, Copenhagen, Denmark).^12^ GBS isolates that tested negative by latex agglutination for all serotypes were typed by PCR using serotype-specific primer sequences.^13^ The presence of antimicrobials in the urine was tested by the disk diffusion method using a sensitive strain of *Bacillus* (*Bacillus subtilis*; ATCC 11774). Briefly, urine was added to a sterile disk placed in the centre of the *B subtilis* inoculated plate. Any zone of inhibition around the disk was considered to be evidence of antimicrobial activity.

Study staff interviewed the participants for demographic, maternal history, and birth history data at the time of enrolment. Mid-upper arm circumference was measured by staff, and the rest of the baseline information was abstracted from medical records. All study specific measurements and sampling were undertaken by trained study staff.

All the enrolled women were contacted by telephone or in person around 28 days after birth to determine the health status of their infant, and to enquire whether the child had been hospitalised for sepsis. If a parent confirmed that their child had been admitted to hospital for any reason, or if a child’s admission to hospital was identified, by passive surveillance, to have been for invasive GBS disease, confirmation of a diagnosis of suspected sepsis or invasive GBS disease was obtained from the medical notes of the attending physician, when available.

### Outcomes

The co-primary outcome, reported here, was the prevalence of rectovaginal GBS among pregnant women. The other co-primary objective was to determine serotype-specific capsular antibody levels in HIV-uninfected pregnant women at term delivery, and will be reported separately.

Secondary GBS colonisation-related outcomes included the vertical transmission rate of GBS and GBS serotype distribution. The sensitivity of LIM broth and direct plating methods for detection of GBS was an exploratory objective. A full list of all prespecified outcomes is provided in the protocol (appendix pp 42–43).

### Statistical analysis

Assuming 15% prevalence of maternal GBS colonisation, we aimed to enrol at least 780 pregnant women at each study site to estimate the prevalence of colonisation with 5% precision (ie, a margin of error of 5% for the 95% CI). Descriptive statistics were presented using counts with proportions for categorical variables and medians with IQRs for quantitative variables. Only participants for whom both maternal and infant colonisation status was known were included in the analysis; mothers who had twins could be included in the analysis if swab data were available from at least one twin.

The sensitivity (detection rate) of each culture method (ie, LIM broth and direct plating) was calculated by taking the proportion of positive samples according to that specific culture method and comparing it with the proportion of samples that were positive according to either the LIM broth or direct plating culture method. Maternal swab specimens were considered to be negative for GBS if no GBS growth was identified on either the vaginal or rectal swab. Conversely, women were categorised as being colonised with GBS if GBS was cultured from either the rectal swab, the vaginal swab, or both. Newborns were categorised as being colonised with GBS if GBS was cultured from any of their swabs (skin surface, rectal, or throat), and negative for GBS in the absence of GBS from all of these sites. Logistic regression analysis and the χ^2^ test were used to determine the association between GBS colonisation and demographic characteristics at enrolment. Multiple logistic regression was used to assess the odds of GBS colonisation according to potential risk factors for colonisation (country, maternal age, presence of antimicrobials in urine, gravida, and mode of delivery). Odds ratios were adjusted for country, age, antimicrobials, gravida, and mode of delivery. Multiple logistic regression analysis was an exploratory hypothesis-generating analysis, and no adjustments were made for multiple testing. A p value of <0·05 was considered significant. Data were analysed using R software (version 4.1.1) and Prism Graphpad Prism (version 8.0.2 9).

### Role of the funding source

The funder of the study had no role in the study design, data collection, data analysis, data interpretation, or writing of the report.

## Results

**Figure 1:**
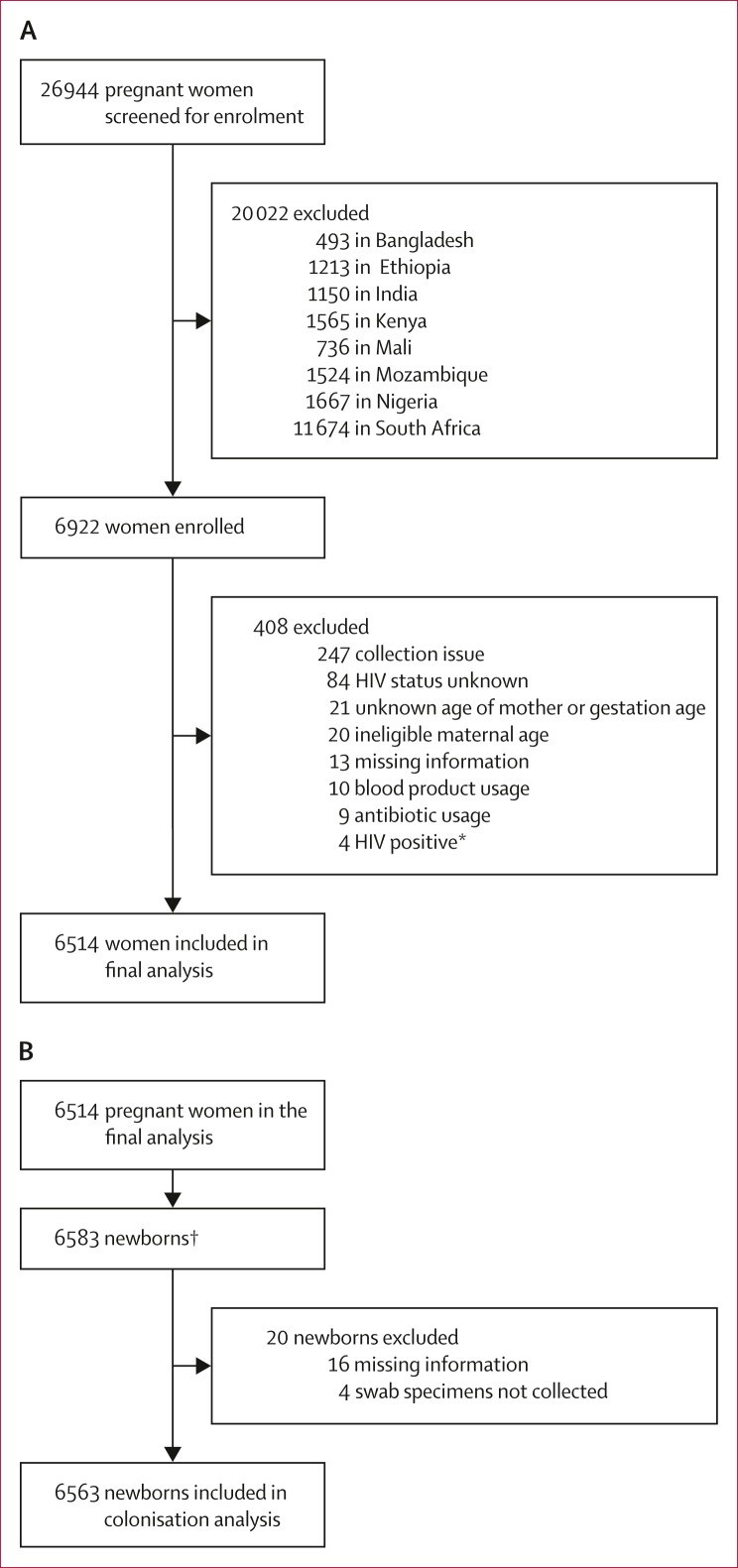
Study profile (A) Pregnant participants. (B) Newborns. Inclusion and exclusion by country is shown in the appendix (pp 8–9). ∗HIV testing was not done at sites where the prevalence of HIV was less than 1% during the study period and not standard of care (ie, Bangladesh). †6445 singleton newborns and 69 pairs of twins were born.

Due to inconsistencies with specimen collection and processing of samples in Bhutan (appendix pp 2–3), data from the Bhutan site were excluded from the final analysis. Overall, 6922 pregnant women were enrolled across the remaining eight countries included in the analysis from Jan 10, 2016, to Dec 11, 2018 (figure 1). The analysed dataset included 6514 (94·1%) of the enrolled women and 6563 of their newborns. Of the 6514 women in the analysed dataset, 69 had twins, with swabs collected from both infants in 53 sets of twins and one infant in 16 sets of twins. The number of participants eligible for analysis ranged from 759 to 892 by country. Reasons for non-inclusion in the final analysis are reported in the appendix (pp 8–9).

The median age of the participants across all sites was 25 years (IQR 22–30), ranging from 22 years (20–27) in Bangladesh to 30 years (27–34) in Nigeria. All the women were of Asian ethnicity in Bangladesh and India, and 96·5–100·0% of the women at the African sites were Black (table 1; appendix pp 10–25). A summary of the proportion of data that was missing is provided in the appendix (p 28).

**Table 1:** Demographics of the study population at time of enrolment

	Overall (N=6514)	Participants with GBS colonisation (N=1572)	Participants without GBS colonisation (N=4942)	p value∗
Median age (IQR), years	25 (22–30); n=6512	26 (22–30); n=1572	25 (21–29); n=4940	0·0015
Age group, years	··	··	··	0·010
<20	1238/6512 (19·0%)	269/1572 (17·1%)	969/4940 (19·6%)	··
20–<25	2148/6512 (33·0%)	506/1572 (32·2%)	1642/4940 (33·2%)	··
25–<30	1835/6512 (28·2%)	436/1572 (27·7%)	1399/4940 (28·3%)	··
30–<35	851/6512 (13·1%)	234/1572 (14·9%)	617/4940 (12·5%)	··
35–<40	378/6512 (5·8%)	109/1572 (6·9%)	269/4940 (5·4%)	··
≥40	62/6512 (1·0%)	18/1572 (1·1%)	44/4940 (0·9%)	··
Race or ethnicity	··	··	··	<0·0001
Black	4759/6427 (74·0%)	1243/1564 (79·5%)	3516/4863 (72·3%)	··
Asian	1640/6427 (25·5%)	312/1564 (19·9%)	1328/4863 (27·3%)	··
Other	28/6427 (0·4%)	9/1564 (0·6%)	19/4863 (0·4%)	··
Median gravidity (IQR)	2 (1–3); n=6485	2 (1–3); n=1572	2 (1–3); n=4913	<0·0001
Gravidity	··	··	··	<0·0001
1	2435/6485 (37·5%)	513/1572 (32·6%)	1922/4913 (39·1%)	··
2	1703/6485 (26·3%)	397/1572 (25·3%)	1306/4913 (26·6%)	··
3	1062/6485 (16·4%)	293/1572 (18·6%)	769/4913 (15·7%)	··
≥4	1285/6485 (19·8%)	369/1572 (23·5%)	916/4913 (18·6%)	··
Median parity (IQR)	1 (1–2); n=3938	2 (1–3); n=1031	1 (1–2); n=2907	0·0012
Parity	··	··	··	0·019
0	343/3938 (8·7%)	80/1031 (7·8%)	263/2907 (9·0%)	··
1	1725/3938 (43·8%)	418/1031 (40·5%)	1307/2907 (45·0%)	··
2	908/3938 (23·1%)	248/1031 (24·1%)	660/2907 (22·7%)	··
3	455/3938 (11·6%)	139/1031 (13·5%)	316/2907 (10·9%)	··
≥4	507/3938 (12·9%)	146/1031 (14·2%)	361/2907 (12·4%)	··
Previous stillbirth	··	··	··	0·63
Yes	217/3714 (5·8%)	61/983 (6·2%)	156/2731 (5·7%)	··
No	3497/3714 (94·2%)	922/983 (93·8%)	2575/2731 (94·3%)	··
Previous pregnancy loss	··	··	··	0·57
Yes	831/3910 (21·3%)	211/1025 (20·6%)	620/2885 (21·5%)	··
No	3079/3910 (78·7%)	814/1025 (79·4%)	2265/2885 (78·5%)	··
Median maternal MUAC (IQR), cm	27 (25–30); n=5633	28 (26–31); n=1274	27 (24–30); n=4359	<0·0001
Chorioamnionitis	··	··	··	0·27
Yes	10/6489 (0·2%)	4/1569 (0·3%)	6/4920 (0·1%)	··
No	6479/6489 (99·8%)	1565/1569 (99·7%)	4914/4920 (99·9%)	··
Single or multiple birth	··	··	··	0·17
Singleton	6445/6514 (98·9%)	1550/1572 (98·6%)	4895/4942 (99·0%)	··
Twin	69/6514 (1·1%)	22/1572 (1·4%)	47/4942 (1·0%)	··
Antimicrobials in urine	··	··	··	<0·0001
Yes	470/6227 (7·5%)	77/1506 (5·1%)	393/4721 (8·3%)	··
No	5757/6227 (92·5%)	1429/1506 (94·9%)	4328/4721 (91·7%)	··
Bacteriuria†	··	··	··	<0·0001
Yes	435/4958 (8·8%)	405/1162 (34·9%)	30/3796 (0·8%)	··
No	4523/4958 (91·2%)	757/1162 (65·1%)	3766/3796 (99·2%)	··
Number of births from which swabs were collected, n	6567‡	1592	4975	··
Mode of delivery§	··	··	··	0·25
Vaginal	5599/6562 (85·3%)	1372/1591 (86·2%)	4227/4971 (85·0%)	··
Caesarean	963/6562 (14·7%)	219/1591 (13·8%)	744/4971 (15·0%)	··
Reason for caesarean¶	··	··	··	>0·99
Emergency	842/963 (87·4%)	192/219 (87·7%)	650/744 (87·4%)	··
Elective	121/963 (12·6%)	27/219 (12·3%)	94/744 (12·6%)	··
Median neonate birthweight (IQR), kg	3·1 (2·8–3·4); n=6545	3·1 (2·8–3·4); n=1588	3·1 (2·8–3·4); n=4957	0·74

Data are n/N (%) unless otherwise specified. Denominators are the overall number of participants (with available data) in the group identified in the column heading. GBS=group B *Streptococcus*. MUAC=mid-upper arm circumference.

∗ p value comparing categorical variables using Fisher’s exact test or χ^2^ test or comparing quantitative values using Wilcoxon rank sum test for the difference between the GBS positive and GBS negative groups.

† Excludes participants from Ethiopia and Mali, since it was not possible to culture their urine samples.

‡ There were 6583 births (6445 singletons and 69 sets of twins), from which 6567 swabs were collected (in 53 sets of twins both had a swab collected, and in 16 sets of twins only one infant had a swab collected).

§ For mode of delivery, the denominator is the total number of births from which swabs were obtained from the newborn, and for which data on mode of delivery were obtained.

¶ For reason for caesarean, the denominator is total number of caesareans.

The sensitivity of GBS culture from vaginal and rectal swabs using the LIM broth and direct plating methods relative to composite culture positivity by either method is detailed in the appendix (p 7). Overall, the proportion of participants with GBS cultured from vaginal swabs (1365 [21·0%, 95% CI 20·0–22·0] of 6514) was higher than the proportion with GBS cultured from rectal swabs (1115 [17·1%, 16·2–18·1] of 6514; p<0·0001); similar findings were observed in the country-specific analysis (full data not shown), except for Bangladesh (99 [12·6%, 10·4–15·1] of 788 *vs* 102 [12·9%, 10·8–15·5] of 788; p=0·82) and Ethiopia (76 [10·0, 8·1–12·4] of 759 *vs* 68 [9·0%, 7·1–11·2] of 759; p=0·22), where GBS isolation from vaginal and rectal swabs was similar.

**Figure 2:**
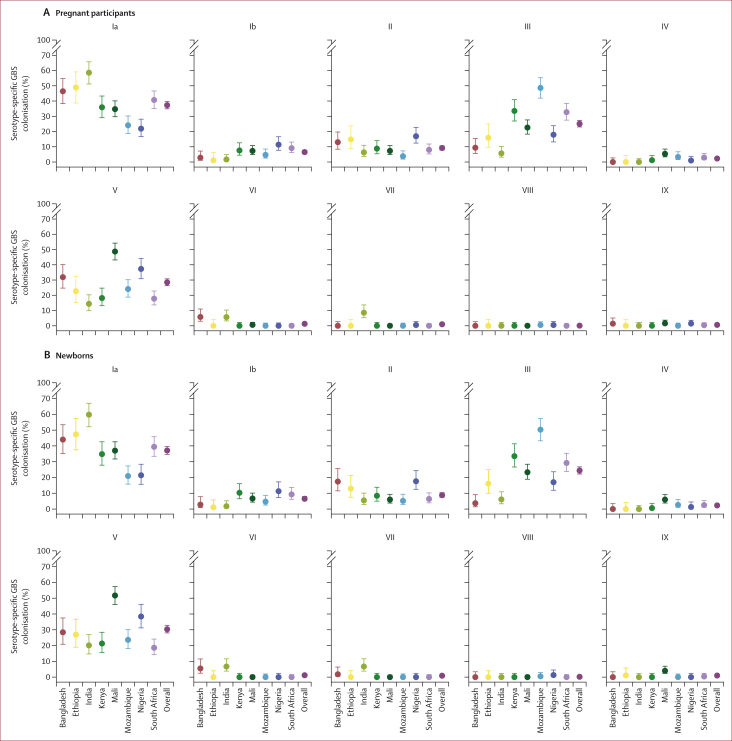
Prevalence of GBS colonisation overall, stratified by culture plating method and by country site (A) Pregnant participants. (B) Newborns. Error bars show 95% CI. GBS=group B *Streptococcus.* LIM broth=Todd–Hewitt broth supplemented with colistin and nalidixic acid.

Overall, the prevalence of maternal GBS colonisation, irrespective of culture method or colonising site, was 24·1% (95% CI 23·1–25·2; 1572 of 6514; figure 2). The prevalence of maternal GBS colonisation was highest in Mali (41·1% [37·7–44·6]; 314 of 764) and lowest in Ethiopia (11·6% [9·5–14·1]; 88 of 759). The prevalence of GBS colonisation in the other African countries was 21·2% (18·5–24·2; 170 of 802) in Kenya, 23·0% (20·3–25·9; 201 of 874) in Nigeria, 27·0% (24·0–30·2; 212 of 785) in Mozambique, and 30·8% (27·9–33·9; 275 of 892) in South Africa. The prevalence of maternal GBS colonisation was 17·5% (15–20·3; 138 of 788) in Bangladesh and 20·5% (17·9–23·3; 174 of 850) in India (figure 2). Overall, the prevalence of GBS colonisation was higher in African countries (25·8%, 24·6–27·1; 1260 of 4876) compared with south Asian countries (19·0%, 17·2–21·1; 312 of 1638; p≤0·0001)

11·0% (95% CI 9·6–12·6; 173 of 1572) of women were colonised with more than one GBS serotype: 10·1% (8·6–11·5; 158 of 1572) had two serotypes and 1·0% (0·5–1·4; 15 of 1572) had three serotypes isolated from rectal or vaginal swabs (or both) and cultured from either the direct plating or LIM broth methods (or both; appendix p 27). Serotypes were concordant between rectal and vaginal swabs in 88·9% (87·2–90·4; 1397 of 1572) of participants. All individual serotypes were included in the analysis of serotype distribution in colonising isolates.

**Figure 3:**
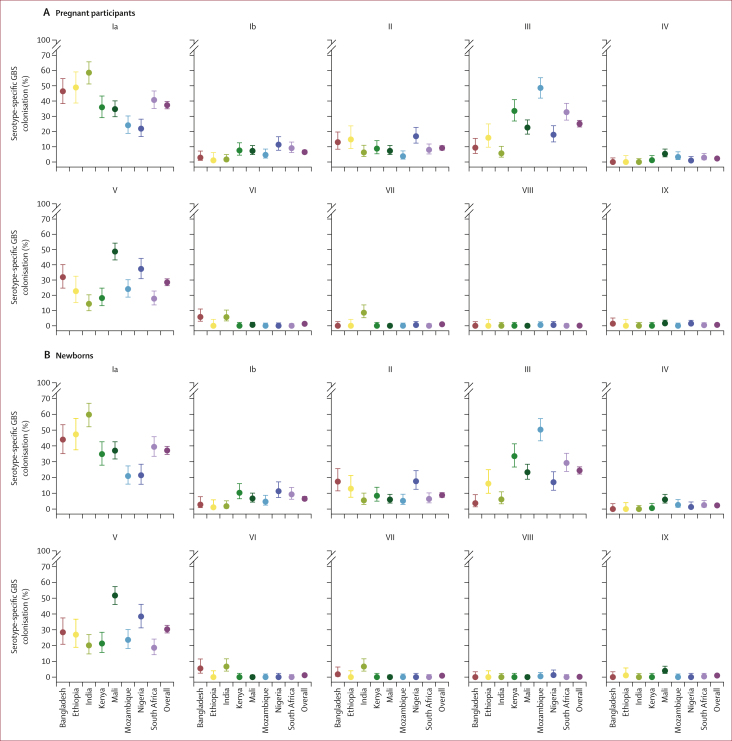
GBS serotype distribution, per country site and overall (A) Pregnant participants. (B) Newborns. Error bars show 95% CI. GBS=group B *Streptococcus.*

Overall, the five most common colonising serotypes were Ia (37·3% [95% CI 34·9–39·7]; 586 of 1572), V (28·5% [26·3–30·8]; 448 of 1572), III (25·1% [23·0–27·3]; 394 of 1572;), II (9·2% [7·8–10·7]; 144 of 1572), and Ib (6·5% [5·4–7·8]; 102 of 1572). Geographical variability was observed in the relative serotype distribution. The proportion of isolates comprising at least one of the five most common colonising serotypes was 1519 (96·6%) of 1572 overall, ranging from 149 (85·6%) of 174 in India to 88 (100·0%) of 88 colonising isolates in Ethiopia. The most common serotypes by country were Ia in Bangladesh, India, Ethiopia, Kenya, and South Africa (ranging from 35·9% [95% CI 29·1–43·3; 61 of 170] in Kenya to 56·8% [51·2–65·7; 102 of 174] in India), serotype V in Mali (48·7% [43·2–54·2]; 153 of 314) and Nigeria (37·3% [30·9–44·2]; 75 of 201), and serotype III in Mozambique (48·6% [41·9–55·3]; 103 of 212; figure 3). Serotype VII comprised 8·6% (5·3–13·7; 15 of 174) of all the isolates in India, where it was the third most common serotype; but was largely absent at sites in other countries. Serotype VI was identified almost exclusively in Bangladesh (5·8% [3·0–11·0; eight of 138) and India (5·7% [3·2–10·3]; ten of 174).

Urine samples from Mali and Ethiopia were deemed not suitable for microbial culturing as they had been frozen after collection, so they were not processed for GBS culture. The overall prevalence of GBS bacteriuria in the other six remaining countries was 8·8% (95% CI [8·0–9·6]; 435 of 4958), ranging from 14·1% (11·9–16·5; 123 of 875) in South Africa to 3·9% (2·8–5·5; 31 of 788) in Bangladesh (appendix p 20). 405 (93·1%) of the 435 women with GBS bacteriuria also had GBS cultured on either vaginal or rectal swabs (table 1). Serotypes were concordant between urine and colonising isolates in 94·1% (91·3–96·0; 381 of 405) of participants (appendix p 28).

We detected antimicrobials in the urine of 470 (7·5%) of 6227 women despite no reporting or record of antibiotics on their antenatal card within the 2 weeks before onset of labour. Presence of antimicrobials in the urine was associated with lower odds of GBS colonisation in the pregnant women (77 [5·1%] of 1506 of women with GBS colonisation had antimicrobials in their urine *vs* 393 [8·3%] of 4721 of women with no GBS colonisation; adjusted odds ratio 0·64 [95% CI 0·49–0·83]; p=0·0008; appendix p 26), with country-specific data in the appendix (pp 10–25). Age, gravida, and mode of delivery did not significantly affect the odds of GBS colonisation (appendix pp 26–27).

With regard to GBS colonisation in newborns, the sensitivity of GBS culture was higher with LIM broth than with direct plating at all sites (figure 2; appendix p 7). GBS colonisation was most common from skin surface samples (17·9% [95% CI 17·0–18·8]; 1172 of 6562), followed by throat swabs (13·7% [12·9–14·5]; 898 of 6562) and rectal swabs (12·8% [12·0–13·6]; 839 of 6563). The overall prevalence of GBS colonisation from any swab site in the newborn was 21·4% (20·5–22·4; 1407 of 6563). The proportion of newborns with colonisation of at least one swab site ranged from 12·3% (10·1–14·8; 93 of 759) in Ethiopia to 38·3% (35–41·8; 300 of 783) in Mali. The overall rate of vertical transmission of GBS from women with rectovaginal GBS colonisation to their newborn was 72·3% (70·0–74·4; 1132 of 1566). GBS vertical transmission rates were similar in Ethiopia, India, Kenya, Mali, and Mozambique, and higher than in Nigeria, South Africa, and Bangladesh, which had the lowest vertical transmission rate (table 2). GBS was also cultured from at least one swab site in 5·2% (4·6–5·9; 257 of 4929) of newborns of mothers with no GBS colonisation. There was a higher prevalence of GBS colonisation in newborns of mothers with GBS bacteriuria (82·4% [78·6–85·7]; 357 of 433) than in newborns of mothers colonised by GBS but without GBS bacteriuria (61·1% [57·6–64·6]; 461 of 754; p<0·0001; appendix p 28).

**Table 2:** Vertical transmission of GBS from the mother to newborn at delivery

	GBS colonisation in newborns of women with GBS rectovaginal colonisation		GBS colonisation in newborns of women without rectovaginal GBS colonisation	
	n/N	% (95% CI)	n/N	% (95% CI)
Bangladesh	77/138	55·8% (47·5–63·8)	32/650	4·9% (95% CI 3·5–6·9)
Ethiopia	67/87	77% (67·1–84·6)	25/667	3·7% (95 CI 2·6–5·5)
India	134/174	77% (70·2–82·6)	30/67	4·4% (95 CI 3·1–6·3)
Kenya	128/169	75·7% (68·8–81·6)	26/630	4·1% (95 CI 2·8–6)
Mali	247/314	78·7% (73·8–82·8)	46/450	10·2% (95 CI 7·8–13·4)
Mozambique	168/212	79·2% (73·3–84·2)	20/573	3·5% (95 CI 2·3–5·3)
Nigeria	125/201	62·2% (55·3–68·6)	31/673	4·6% (95 CI 3·3–6·5)
South Africa	186/271	68·6% (62·9–73·9)	47/610	7·7% (95 CI 5·8–10·1)
Overall	1132/1566	72·3% (70·0–74·4)	257/4929	5·2% (95 CI 4·6–5·9)

GBS=group B *Streptococcus*.

Of the newborns with GBS colonisation, 11·2% (95% CI 9·7–13·0; 158 of 1405) were colonised with two or more serotypes. The highest prevalence of colonisation by more than one GBS serotype among newborns colonised with GBS was in Mali (28·0%, 23·1–33·5; 84 of 300), and colonisation with more than one serotype was lowest in Bangladesh (3·7% [ 1·2–9·7]; four of 109; appendix p 27). The GBS serotype distribution in newborns, expectantly, was similar to their mothers (figure 3). Among 49 sets of twins, both babies were colonised in 13 (27%) sets, whereas only the first-born twin was colonised in four (8%) sets, and only the second-born in three (6%) sets. Among the 13 sets of twins in whom both twins were colonised, GBS serotypes differed between each twin in four (30%) pairs.

20 (0·3%) of 6514 women had a stillbirth, of whom six (30% [95% CI 12·8–54·3]) were colonised with GBS. 19 (0·3%) of 6567 newborns had died at the day-28 follow-up visit across all the study sites (three in Ethiopia, five each from Mali and Nigeria, and six in Mozambique), of whom six (32%) had been colonised by GBS. Overall, 86 cases of suspected neonatal sepsis diagnosed by the attending physician were recorded, including three each in India and Mozambique, 13 in South Africa, and 67 episodes in Kenya. There were three confirmed cases of invasive GBS disease, including one case of early-onset disease in Kenya in a 1-day-old baby (GBS colonisation had not been detected in the newborn at birth or the mother during labour), yielding an incidence of 1·25 (95% CI 0·22–7·01) per 1000 livebirths in Kenya. There were two cases of late-onset disease by day 28, which occurred at age 11 days and age 26 days in two infants in Mozambique; no serotyping was available for the invasive isolates. Neither of these two infants’ mothers were colonised at delivery, but one newborn was found to have been colonised by serotype III at the time of birth.

## Discussion

Our study findings showed the variability in the prevalence of GBS rectovaginal colonisation in pregnant women across different low-income and middle-income African and south Asian countries. Nevertheless, the between-country differences in prevalence of GBS colonisation observed in our study are of a lower magnitude than in reports from similar countries included in a systematic review and meta-analysis by Russell and colleagues,^5^ which recognised differences in sampling and culture methods used in the studies as being limitations in the comparability of interstudy prevalence. Use of a standardised methodological approach across all countries in our study enabled a more robust intercountry comparison of the prevalence of maternal GBS rectovaginal colonisation and vertical transmission across eight different African and south Asian countries. We found that the LIM broth GBS culture method had a higher sensitivity than direct plating for maternal vaginal samples and newborn skin surface, throat, and rectal samples, which illustrates how differences in culture methods, among other variables, could affect the evaluation of the prevalence of GBS colonisation.

The overall prevalence of maternal GBS colonisation in our study of eight LMICs (24·1%, 95% CI 23·1–25·2) is higher than that reported in Russell and colleagues’ global meta-analysis, which included data from 85 countries (18%, 17–19).^5^ Furthermore, the variability of GBS colonisation prevalence in women across the six different African countries (11·6–41·1%) and across the two south Asian countries (17·5–20·5%) was also less than was reported by Russell and colleagues (2–36% across selected African countries and 3–25% across two Indian sites).^5^ At an individual country level, we observed a higher prevalence of maternal GBS colonisation compared with previous studies in India (20·5% in our study *vs* 7·6% in a previous study)^14^ and similarly in Kenya (21·2% *vs* 11·1%);^15^ whereas we observed a similar prevalence of GBS colonisation compared with previous studies for Mozambique,^16^ South Africa,^17^ Bangladesh,^8^ and Ethiopia.^18^ An outlier was Nigeria, where prevalence of GBS colonisation in our study (23·0%) was lower than reported from the same region but in a different study setting (34·2%).^19^ Another notable finding of our study was the extraordinarily high prevalence of maternal GBS rectovaginal colonisation in Mali (41·1%), for which there are no previously published studies. The higher overall prevalence of GBS colonisation in our study compared with previous reports is probably due to a more standardised approach used to investigate GBS colonisation, including separate sampling of rectal and vaginal swabs and the use of LIM broth medium.

The use of intrapartum antibiotic prophylaxis targeted at women colonised with GBS beyond 32 weeks of gestation reduces the risk of early-onset invasive GBS disease by 80%.^20^ Nevertheless, screening for GBS rectovaginal colonisation during pregnancy and implementation of intrapartum antibiotic prophylaxis is not practised in most LMICs due to logistical and cost constraints.^20^ Therefore, maternal vaccination against GBS is likely to be a more feasible option for reducing invasive GBS disease. Determining the serotype distribution, as done in our study, could guide development of a GBS vaccine to provide optimal serotype coverage, at least in relation to early-onset disease.^21^ Our findings on the dominant GBS serotypes across different settings were similar to serotype distribution data of maternal colonising isolates reported in Russell and colleagues’ meta-analysis.^5^ Serotypes Ia, Ib, II, III, and V were the most common serotypes across all the African sites, whereas serotypes VII and VI were among the top five serotypes in the south Asian sites only. A study from Sri Lanka also reported serotype VI as a common maternal colonising serotype (17·9% of all isolates).^22^ Furthermore, a study in a south Indian population found that 8·6% of isolates implicated in early-onset disease were serotype VI and 6·9% were serotype VII,^23^ indicating the clinical relevance of these serotypes in pregnant individuals with GBS colonisation. The reasons for geographical variability in serotype distribution among African and south Asian women warrants further investigation.

The current hexavalent (serotypes Ia, Ib, II, III, IV, and V) GBS polysaccharide–protein conjugate vaccine (GBS6) being evaluated in pregnant women^21^ includes serotypes that comprised 96·6% of all maternal colonising isolates in our study, ranging from 100·0% in Ethiopia and Kenya to 85·8% in India. It has been shown that higher serotype-specific IgG in a newborn, acquired through transplacental transfer, is associated with reduced risk of invasive GBS disease and a lower risk of acquisition of GBS rectovaginal colonisation in the newborn’s mother during pregnancy.^24^^,^^25^ Consequently, although vaccination of pregnant individuals with a GBS6 vaccine could reduce colonisation by the serotypes in the vaccine, there could be replacement colonisation by non-vaccine serotypes such as serotype VI and VII, especially in regions where those serotypes already prevail.

Our study also identified a high vertical GBS transmission rate from women colonised by GBS to their newborns in all countries (ranging from 55·8% in Bangladesh to 79·2% in Mozambique). Earlier studies reported considerable variation within and between geographical regions with regard to vertical transmission of GBS, ranging from 7·6% in China to 63·3% in Ethiopia.^7^^,^^8^^,^^19^ The high vertical transmission of GBS observed in our study could be due to greater sensitivity in detecting GBS by sampling three different areas in the newborn, as well as the culture method we used. An oddity in our findings from Ethiopia was the slightly higher prevalence of newborn GBS colonisation (12·3%) compared with the prevalence of rectovaginal colonisation in their mothers (11·6%). The findings from Ethiopia (and Bhutan, which was excluded from the analysis) suggest that the maternal rectovaginal swabbing methods (all culturing for the Ethiopia site was done in South Africa) might have been suboptimal, despite use of standardised operating procedures and training, and repeated attempts at quality assurance, which was implemented across all the study sites. Consequently, we might have inadvertently underestimated the prevalence of maternal GBS colonisation in Ethiopia. We also observed that 5·2% of newborns colonised with GBS were born to women in whom no GBS colonisation was identified, which was evident across all sites. Consequently, there might have been some under-ascertainment of GBS rectovaginal colonisation in participants, if we assume that mothers are the sole source of GBS transmission to newborns at the time of birth. As collection of rectovaginal swabs is subject to operator competency, there might be inadvertent underestimation of GBS colonisation in women, even when training is provided and there are standard operating procedures as was the case in our study.

The prevalence of GBS colonisation in newborns of mothers with GBS bacteriuria was significantly higher than in newborns of mothers colonised by GBS but without GBS bacteriuria. Maternal GBS bacteriuria has been associated with high density of vaginal GBS colonisation in women and an increased risk of early-onset invasive GBS disease in their newborns.^26^, ^27^, ^28^ The scarcity of diagnostic facilities for blood culture remains a major challenge in delineating the causes of bacterial sepsis in low-income countries. Consequently, the burden of invasive GBS disease is probably underestimated and difficult to ascertain in these settings. We identified only one case of early-onset invasive GBS disease, yielding an incidence of 1·25 per 1000 livebirths in Kenya, although it is worth noting that investigation of invasive GBS disease was not undertaken systematically as part of the study and was dependent on the standard-of-care practices at the site. Evaluating the vaccine efficacy of GBS6 against serotype-specific bacteriuria in pregnant women should be considered as a potential exploratory objective that could indirectly infer efficacy of GBS6 against early-onset disease.

Limitations of our study include that it was designed to determine the prevalence of GBS colonisation and did not systematically investigate invasive GBS disease, as we relied on standard of care to identify invasive cases. Additionally, we only followed up participants to day 28, so we would not have been able to document cases of late-onset invasive disease that occurred between day 29 and day 90. Therefore, although only one case of early-onset invasive GBS disease was confirmed in our cohort of neonates, the true incidence is likely to have been higher based on the previous estimate that 1–2% of babies born to women with rectovaginal GBS colonisation develop invasive GBS disease in the absence of intrapartum antibiotic prophylaxis.^3^ Participants were mainly enrolled from a single study centre in each country, so the findings might not be generalisable to the wider population in that country. Another limitation was that GBS colonisation was reported qualitatively (ie, as either being present or not), which precluded a quantitative assessment of the density of GBS rectovaginal colonisation and association with bacteriuria in the mother or association with rate of vertical transmission to the newborn. Furthermore, as only a single colony was used for serotyping per sample, we probably underestimated the frequency of colonisation by multiple GBS serotypes. At the Ethiopian and Malian sites, swab specimens were shipped in STGG to South Africa to be processed, which could have introduced bias with respect to site-specific prevalence; however, considering that Ethiopia and Mali were the African countries with the lowest and highest prevalence of maternal colonisation, respectively, there is no suggestion of the potential bias being in a single direction. The method of assessing the health status of infants was either in person or via telephone interview at all sites. Hospital records were also used to review cause of hospitalisation and invasive GBD cases when required, and these records might have varied among sites, which could have introduced variation in data collection and reporting. Although there were multiple comparisons undertaken in the analyses, no adjustment to p values were made for this observational study, warranting caution in interpretation of the comparative analysis. Nevertheless, in our analyses, all statistically significant p values were less than 0·001, indicating the strong likelihood of an association not being due to chance.

In conclusion, using a standardised approach to sampling and standardised methods for GBS culture, our study reported a high prevalence of GBS colonisation in most study settings, with some geographical variability within African countries. Post-licensure vaccine effectiveness studies should focus on maternal GBS serotype distribution, because non-vaccine serotype replacement could occur in response to vaccine immune pressure. The high prevalence of maternal GBS colonisation in our study settings indicates that early-onset invasive GBS disease is likely to be prevalent in the study settings, particularly in the absence of any screening for GBS colonisation in women during pregnancy and no systematic use of intrapartum antibiotic prophylaxis.

## Data sharing

De-identified individual-level participant data and data dictionary will be available for sharing after approval of a proposal by the University of the Witwatersrand Human Ethics Research Committee and following a signed data access agreement. Requests for data sharing can be directed to the corresponding author.

## Declaration of interests

SAM declares grant funding to his institution for research on GBS, separate from this study, from Pfizer, GlaxoSmithKline, and Minervax. GK declares grant funding to his institution for research on GBS from the Bill & Melinda Gates Foundation and PATH. All other authors declare no competing interests.
