# Supplementary material for: Prevalence of group B Streptococcus colonisation in mother–newborn dyads in low-income and middle-income south Asian and African countries: a prospective, observational study
Source: Lancet Microbe. 2024 Oct;5(10):None. doi: 10.1016/S2666-5247(24)00129-0 (PMC11464403; doi:10.1016/S2666-5247(24)00129-0)
Supplement: Supplementary appendix [file mmc1.pdf]

THE LANCET
Microbe

Supplementary appendix

This appendix formed part of the original submission and has been peer reviewed. We post it as supplied by the authors.

Supplement to: Kwatra G, Izu A, Cutland C, et al. Prevalence of group B *Streptococcus* colonisation in mother–newborn dyads in low-income and middle-income south Asian and African countries: a prospective, observational study. *Lancet Microbe* 2024. https://doi.org/10.1016/S2666-5247(24)00129-0

Supplementary information for "Prevalence of Group B Streptococcus colonization in mother-newborn dyads in low and middle income South Asian and African countries: A prospective, observational cross sectional study."

Contents

Study sites and Population Characteristics 2

Bhutan Site Exclusion from Colonization analysis 2

Training at VIDA 3

External Quality Assessment 4

Inclusion and Exclusion Criteria 5

Specimen collection and processing 5

Data Processing, management and storage 6

Comparison of GBS culture by direct plating and selective broth 7

References 28

Protocol 29

Study sites and Population Characteristics

Supplementary Table 1: Study sites and Population Characteristics

Country	Recruitment Period	Recruitment facility	Population Characteristics
Bangladesh	20 April 2016 - 19 May 2017	Kumudini Women's Medical College Hospital, Mirzapur	Rural
Bhutan	02 January 2017- 29 October 2017	Jigme Dorji Wangchuk National Referral Hospital (JDWNRH), Thimphu	Urban and rural both
India	02 May 2017 - 20 November 2017	Christian Medical College Hospital, Vellore	Urban predominance, some women from rural areas
Ethiopia	19 June 2017- 09 October 2018	Adama Hospital Medical College, Adama City	Urban and rural both.
Kenya	10 January 2016 – 24 October 2016	Kilifi County Hospital, Kilifi	Rural
Mali	12 February 2018 - 21November 2018	The community health center (ASACOBA), Bamaku<br><br>CSREF 1 (Referal Health Center of commune 1), Bamaku	Urban
Mozambique	27 December 2016- 30 January 2018	Manhiça district hospital, Manhica	Rural
Nigeria	05 April 2016 – 28 August 2017	Abuja Teaching Hospital, Gwagwalada	Urban and rural population
South Africa	03 October 2017 – 11 December 2018	Chris Hani Baragwanath Academic Hospital, Soweto<br><br>Liliyan Ngoyi community clinic, Soweto	Urban

Bhutan Site Exclusion from Colonization analysis

In the interim preliminary analysis (at 50% of the sample size) very low colonization prevalence was reported from Bhutan compared to other sites. To quality control their specimen collection process

and microbiological culture methods, duplicate vaginal swab specimens were collected for 46 participants and were shipped to South Africa (SA) for culturing processing. The duplicate vaginal swabs were processed immediately in the laboratory at SA. The GBS positivity rate was highly discordant between Bhutan (4.5%) and South Africa (29.5%) laboratories. In addition, random 48 duplicate swabs specimens (Mix of maternal Rectal, Infant throat, infant rectal and infant skin) were also collected and shipped to South Africa for processing. The GBS positivity results were highly discordant between two laboratories (Bhutan (0%) and South Africa (16.7%).

Supplementary Table 2: Comparison of GBS colonization rates for Bhutan swab specimens at Bhutan and South Africa (Wits-Vida) laboratory.

	Vaginal Swabs (n=44)	Swabs (Maternal Rectal, Infant throat, Infant rectal and Infant skin) (n=48)	Overall (n=92)
Bhutan	2 (4.5%)	0 (0%)	2 (2.2%)
South Africa	13 (29.5%)	8 (16.7%)	21 (22.8%)

Retraining and onsite visit was performed at Bhutan site on specimen and culturing process but no improvement in GBS colonization was noted. The results were presented to study scientific advisory committee, and it was decided not to further process swabs at Bhutan site and exclude Bhutan site from colonization analysis due to sub-optimal processing and GBS culturing process.

Training at VIDA

Two Laboratory scientists and clinical staff from all the participating sites received training applicable to their responsibilities within 8 to 10 weeks prior to the start of enrolment at the site. Training included methods of specimen collection, data capturing and GBS culture and identification. The training was conducted in phases at Wits-VIDA to ensure that attendees from each study site received close supervision. This was complemented by the study lead (GK) undertaking visits to each of the sites immediately prior to enrolment to provide onsite assistance and training to other study-staff

members, and evaluate the site readiness (laboratory and clinical) to initiate the study. Furthermore, additional intermittent (2-3 per site) site visits were undertaken by GK to compliance with the protocol and procedures. External quality assessment (EQA) was performed prior to the initiation of clinical sample collection for each site. .

External Quality Assessment

External Quality assessment (EQA) was performed on GBS culturing, isolation, and identification prior to the initiation of the collection of clinical samples. This was done as follows:

- A materials pack was shipped to each study site, and included:
    - 20 EQA samples which consist of lyophilised mixtures of normal vaginal and rectal flora that are either positive or negative for GBS.
    - All laboratory reagents and culture media required to process these samples.
- Site laboratories were given two weeks to e-mail their GBS isolation and identification results from the EQA samples.
- A pass mark of 90% concordance was considered acceptable.
- Sites which fail to achieve this pass mark were re-evaluated as follows:
    - Problem samples were investigated to determine the cause of the errors. For these purposes, site laboratories were encouraged to take photographs of the plates to assist us in resolving issues.
    - A site laboratory was approved if the discordant results were easily resolved telephonically.

- Site laboratories with low scores that cannot be approved through minor procedural steps were visited by a representative from the VIDA laboratory for further training and approval.

Inclusion and Exclusion Criteria

Inclusion criteria included pregnant women age 18 to 45 years age, delivery at ≥37 weeks gestation age based on date of the last menstrual period and corroborated by physical exam, or ultrasound examination if available and documented to be HIV-uninfected prior to study-enrolment. Testing of women for HIV-1 was not undertaken in Bangladesh, as the population prevalence of maternal HIV-1 was less than one percent at the time of the study[1] . The study exclusion criteria included reported use of antibiotics in the two weeks prior to delivery, any acute illness, symptomatic vaginal discharge and a known or suspected condition in which vaginal examinations and swab collection was contradicted, inability to obtain maternal blood or blood transfusion in the 30 day period before delivery. Although one of the inclusion criteria was "No Antibiotics treatment in the two weeks prior to delivery", due to the possibility of mis-reporting by the participants, we decided to add an additional layer of screening to ensure that the participant did not have any antimicrobial activity, which could have influenced the sensitivity of identifying GBS by culture.

Specimen collection and processing

Participants were screened for enrolment during the early stages of labor. Consenting and eligible women were allocated a unique identification number, following which demographic and pregnancy related data was collected. All sampling was undertaken by study staff. Separate lower vaginal and rectal swabs were collected from the women after the onset of labor, and preferably prior to rupture of amniotic sac membranes. Separate skin (surface swabs of the umbilicus, outer ear and axillary fold), rectal and throat swabs were obtained from the baby prior to cleansing or bathing. The swabs were collected using rayon-tipped swabs (MW170, Medicalwire, UK) that were placed into separate

Amies transport mediugm without charcoal. Swabs were transported on ice to the site laboratory and processed within 24 hours of collection for GBS detection and isolation. In addition, maternal urine sample were processed for GBS culture and presence for antimicrobials.

Swabs were processed onsite laboratories according to standard Centre for Diseases Control and Prevention (CDC, USA) guidelines [1]. Briefly, swabs were inoculated onto selective media ((CHROMagar StrepB; CA; Direct Plating (DP)) and into selective broth (Todd-Hewitt broth supplemented with colistin and nalidixic acid; Lim broth; BD, catalogue number# 296266). The selective broth was further subcultured onto selective media (Selective Broth method, SB). Urine samples (10ul) were directly plated on onto selective media for culturing. A cut-off of ≥10000 CFU/mL was used to define bacteriuria. GBS-like colonies were isolated and confirmed as GBS by testing for Christie Atkins Munch-Petersen (CAMP) factor, inability to hydrolyze esculin, catalase negativity and group B antigen detection. For two sites (Ethiopia and Mali) swab specimens were stored in 1.0ml STGG storage medium and shipped to Wits-VIDA for GBS culturing, due to logistics issues associated with supply of consumables and reagents to the sites. Briefly, STGG medium containing swab was thoroughly vortexed and 100ul was plated on CA and remaining 900 ul of STGG medium with swab was inoculated into selective broth.

Data Processing, management and storage

Data was collected on study-specific data collection forms and entered into study-specific secure online databases at each study site. Collection forms and databases were uniform across sites. Data was transferred to Wits-VIDA monthly. Internal monitoring and quality assurance was conducted on key variables at each site by the local data teams. On a regular basis the study site data manager, generated a set of data listings that was reviewed by the Wits-VIDA coordinating site. Aggregated data from all the sites was compiled, quality checked, stored and analysed at Vida.

Results supplementary material:

Comparison of GBS culture by direct plating and selective broth

In pregnant women:

The culture of GBS from the vaginal swabs was higher using the SB (18.1%; 1177/6504; 95%CI:17.2-19.1), than the DP (16.1%; 1048/6510; 95%CI:15.2-17.0 p=0.003) culture method. Relative to the composite of culture positivity by either method, the sensitivity for culture of GBS was 86.2% (95%CI: 84.3-88.0) and 76.8% (95%CI:74.5-78.9) for the SP and DP methods, respectively. There was no difference in GBS culture positivity between the SB (13.9%; 901/6502; 95%CI: 13.0-14.7) and DP (13.3%; 867/6505; 95%CI 75.2-80.1; p=0.384) methods for rectal swabs. For rectal swabs, the sensitivity of SB and DP were 80.8% (95%CI: 78.4-83.0) and 77.8% (95%CI: 75.2-80.1, respectively, relative to culture positivity by either method, Figure 1a.

In newborns:

Overall, the culture of GBS in newborns was higher on SB compared with DP plating, including for skin surface (15.5%, 95%CI 14.6-16.4; 1014/6552 vs. 12.6%, (95%CI 11.8-13.4; 827/6558); p<0.001), rectal (11.6%, 95%CI 10.9-12.4; 758/6516] vs. 7.1%, 95%CI 6.5-7.7; 465/6551; p<0.001) and throat (12.5%, 95%CI 11.7-13.3; 815/6523) vs. 7.7%, 95%CI 7.1-8.3; 503/6555; p<0.001) samples . Similar observations were evident for all sites except in Nigeria, where skin swabs sampled by SB (8.1%, 72/885; 95%CI: 6.5-10.1) had a lower yield of isolating GBS compared with DP method (11.3%, 101/886; 95%CI: 9.4-13.6; p=0.021). Compared with the composite of positivity from either SB or DP, the sensitivity of SB was 91.4% (1293/1414; 95%CI: 90.1-92.8) and 68.4% (968/1414; 95%CI: 66.0-70.8) for DP; Figure 1b.

Supplementary Table 3a: Maternal Participant enrolled in the study and reasons for non-inclusion in the final analysis.

	Bangladesh	Ethiopia	India	Kenya	Mali	Mozambique	Nigeria	South Africa	Overall
Participant screened	1284	2196	2000	2374	1582	2351	2548	12609	26944
Participant recruited	791	983	850	809	846	827	881	935	6922
Maternal Age ineligibility	3	3		5		3	3	3	20
Sample collection/Process ineligibility		196		1	3	26		21	247
Blood product usage/Missing		4		1		2	2	1	10
Antibiotic usage								9	9
HIV Positive*		2				1	1		4
HIV Unknown		8			74			2	84
Ineligible /Missing gestation age/Missing mother age		5			4	7	1	4	21
Missing multiple information		6			1	3		3	13
Final Eligible participant	788	759	850	802	764	785	874	892	6514

*HIV testing was not done at sites where prevalence of HIV was < 1% at the time study enrolment and not standard of care (Bangladesh).

Supplementary Table 3b: Enrolled maternal participants newborns and reasons for non-inclusion in the final analysis

	Bangladesh	Ethiopia	India	Kenya	Mali	Mozambique	Nigeria	South Africa	Overall
Maternal Participant	788	759	850	802	764	785	874	892	6514
Singletons	782	744	848	801	743	779	863	887	6445
Twins	6	15	2	1	21	6	11	7	69
Overall babies born	794	774	852	803	785	791	885	901	6583
Missing multiple information		14	2						16
Swab specimens missed		1		1	2				4
Newborn for colonization analysis	794	759	850	802	783	791	885	901	6563

Supplementary Table 4a: Demographics of the study population at Bangladesh at the time of enrolment.

	**Overall (N = 788)**	**Participants with GBS colonisation (N=138)**	**Participants without GBS colonisation (N= 650)**	**p value***
Median age (IQR), years	22 (20-27) N=787	24 (20-27) N=138	22 (20-27) N=649	0.15
Age group, years				0.6
<20	303/787 (38.5%)	46/138 (33.3%)	257/649 (39.6%)	
20–<25	246/787 (31.3%)	46/138 (33.3%)	200/649 (30.8%)	
25–<30	192/787 (24.4%)	36/138 (26.1%)	156/649 (24.0%)	
30–<35	38/787 (4.8%)	8/138 (5.8%)	30/649 (4.6%)	
35–<40	8/787 (1.0%)	2/138 (1.4%)	6/649 (0.9%)	
≥40	0/787 (0.0%)	0/138 (0.0%)	0/649 (0.0%)	
Race or ethnicity				
Black	0/787 (0.0%)	0/138 (0.0%)	0/649 (0.0%)	
Asian	787/787 (100.0%)	138/138 (100.0%)	649/649 (100.0%)	
Other	0/787 (0.0%)	0/138 (0.0%)	0/649 (0.0%)	
Median gravidity (IQR)	2 (1-2) N=788	2 (1-2) N=138	2 (1-2) N=650	0.45
Gravidity				0.065
1	379/788 (48.1%)	61/138 (44.2%)	318/650 (48.9%)	
2	231/788 (29.3%)	43/138 (31.2%)	188/650 (28.9%)	
3	127/788 (16.1%)	30/138 (21.7%)	97/650 (14.9%)	
≥4	51/788 (6.5%)	4/138 (2.9%)	47/650 (7.2%)	
Median parity	1 (1-2) N=409	1 (1-2) N=77	1 (1-2) N=332	0.78
Parity				0.95
0	54/409 (13.2%)	9/77 (11.7%)	45/332 (13.6%)	
1	233/409 (57.0%)	47/77 (61.0%)	186/332 (56.0%)	
2	100/409 (24.4%)	18/77 (23.4%)	82/332 (24.7%)	
3	20/409 (4.9%)	3/77 (3.9%)	17/332 (5.1%)	
≥4	2/409 (0.5%)	0/77 (0.0%)	2/332 (0.6%)	
Previous stillbirth				0.31
Yes	29/409 (7.1%)	8/77 (10.4%)	21/332 (6.3%)	
No	380/409 (92.9%)	69/77 (89.6%)	311/332 (93.7%)	
Previous pregnancy loss				0.31
Yes	78/409 (19.1%)	11/77 (14.3%)	67/332 (20.2%)	
No	331/409 (80.9%)	66/77 (85.7%)	265/332 (79.8%)	
Median maternal MUAC (IQR)	27 (24-30) N=788	28 (25-30) N=138	27 (24-30) N=650	0.38
Chorioamnionitis				
Yes	0/788 (0.0%)	0/138 (0.0%)	0/650 (0.0%)	
No	788/788 (100.0%)	138/138 (100.0%)	650/650 (100.0%)	
Single or multiple birth				>0.99
Singleton	782/788 (99.2%)	137/138 (99.3%)	645/650 (99.2%)	
Twin	6/788 (0.8%)	1/138 (0.7%)	5/650 (0.8%)	

Antimicrobials in urine				0.24
Yes	62/788 (7.9%)	7/138 (5.1%)	55/650 (8.5%)	
No	726/788 (92.1%)	131/138 (94.9%)	595/650 (91.5%)	
Bacteriuria				<0.0001
Yes	31/788 (3.9%)	31/138 (22.5%)	-	
No	757/788 (96.1%)	107/138 (77.5%)	650/650 (100.0%)	
Total number of births	794	139	655	
Mode of delivery				
Vaginal	589/794 (74.2%)	98/139 (70.5%)	491/655 (75.0%)	
Caesarean	205/794 (25.8%)	41/139 (29.5%)	164/655 (25.0%)	
Emergency	205/205 (100.0%)	41/41 (100.0%)	164/164 (100.0%)	
Elective	0/205 (0.0%)	0/41 (0.0%)	0/164 (0.0%)	
Median birthweight of neonate in kilograms (IQR)	2.8 (2.5-3.1) N=794	2.8 (2.5-3.1) N=139	2.8 (2.5-3.1) N=655	0.82

Data are n/N (%) unless otherwise specified. GBS=group B *Streptococcus*. MUAC=mid-upper arm circumference. Denominator is the overall number of participants (with available data) in the group identified in the column heading. For mode of delivery, the denominator is the total number of births. *p value comparing categorical variables using Fisher exact test or χ^2 test or comparing quantitative values using Wilcoxon rank sum test for the difference between the GBS positive and GBS negative groups.

Supplementary Table 4b: Demographics of the study population at Ethiopia at time of enrolment

	**Overall (N = 759)**	**Participants with GBS colonisation (N=88)**	**Participants without GBS colonisation (N= 671)**	**p value***
Median age (IQR), years	25 (22-28) N=758	23.5 (20.75-28) N=88	25 (22-28) N=670	0.11
Age group, years				0.23
<20	137/758 (18.1%)	22/88 (25.0%)	115/670 (17.2%)	
20–<25	320/758 (42.2%)	37/88 (42.0%)	283/670 (42.2%)	
25–<30	230/758 (30.3%)	21/88 (23.9%)	209/670 (31.2%)	
30–<35	52/758 (6.9%)	8/88 (9.1%)	44/670 (6.6%)	
35–<40	16/758 (2.1%)	0/88 (0.0%)	16/670 (2.4%)	
≥40	3/758 (0.4%)	0/88 (0.0%)	3/670 (0.4%)	
Race or ethnicity				
Black	678/678 (100.0%)	81/81 (100.0%)	597/597 (100.0%)	
Asian	0/678 (0.0%)	0/81 (0.0%)	0/597 (0.0%)	
Other	0/678 (0.0%)	0/81 (0.0%)	0/597 (0.0%)	
Median gravidity (IQR)	1 (1-2) N=737	1 (1-2) N=88	1 (1-2) N=649	0.71
Gravidity				0.87
1	417/737 (56.6%)	52/88 (59.1%)	365/649 (56.2%)	
2	162/737 (22.0%)	18/88 (20.5%)	144/649 (22.2%)	
3	91/737 (12.3%)	9/88 (10.2%)	82/649 (12.6%)	
≥4	67/737 (9.1%)	9/88 (10.2%)	58/649 (8.9%)	
Median parity	1 (1-2) N=334	1 (1-2) N=36	1 (1-2) N=298	0.65
Parity				0.47
0	48/334 (14.4%)	6/36 (16.7%)	42/298 (14.1%)	
1	160/334 (47.9%)	15/36 (41.7%)	145/298 (48.7%)	
2	78/334 (23.4%)	8/36 (22.2%)	70/298 (23.5%)	
3	26/334 (7.8%)	2/36 (5.6%)	24/298 (8.1%)	
≥4	22/334 (6.6%)	5/36 (13.9%)	17/298 (5.7%)	
Previous stillbirth				>0.99
Yes	9/323 (2.8%)	1/36 (2.8%)	8/287 (2.8%)	
No	314/323 (97.2%)	35/36 (97.2%)	279/287 (97.2%)	
Previous pregnancy loss				0.76
Yes	29/323 (9.0%)	2/36 (5.6%)	27/287 (9.4%)	
No	294/323 (91.0%)	34/36 (94.4%)	260/287 (90.6%)	
Median maternal MUAC (IQR)	24 (22-25) N=741	24.35 (22-25.075) N=86	24 (22-25) N=655	0.38
Chorioamnionitis				
Yes	0/748 (0.0%)	0/88 (0.0%)	0/660 (0.0%)	
No	748/748 (100.0%)	88/88 (100.0%)	660/660 (100.0%)	
Single or multiple birth				0.69
Singleton	744/759 (98.0%)	86/88 (97.7%)	658/671 (98.1%)	
Twin	15/759 (2.0%)	2/88 (2.3%)	13/671 (1.9%)	

Antimicrobials in urine				0.49
Yes	109/748 (14.6%)	15/85 (17.6%)	94/663 (14.2%)	
No	639/748 (85.4%)	70/85 (82.4%)	569/663 (85.8%)	
Bacteriuria				
Yes	-	-	-	
No	-	-	-	
Total number of births	760	88	672	
Mode of delivery				
Vaginal	657/755 (87.0%)	77/87 (88.5%)	580/668 (86.8%)	
Caesarean	98/755 (13.0%)	10/87 (11.5%)	88/668 (13.2%)	
Emergency	98/98 (100.0%)	10/10 (100.0%)	88/88 (100.0%)	
Elective	0/98 (0.0%)	0/10 (0.0%)	0/88 (0.0%)	
Median birthweight of neonate in kilograms (IQR)	3.3 (3-3.5) N=751	3.3 (3-3.5) N=88	3.2 (3-3.5) N=663	0.63

Data are n/N (%) unless otherwise specified. GBS=group B *Streptococcus*. MUAC=mid-upper arm circumference. Denominator is the overall number of participants (with available data) in the group identified in the column heading. For mode of delivery, the denominator is the total number of births. *p value comparing categorical variables using Fisher exact test or χ^2 test or comparing quantitative values using Wilcoxon rank sum test for the difference between the GBS positive and GBS negative groups.

Supplementary Table 4c: Demographics of the study population at India at time of enrolment

	**Overall (N = 850)**	**Participants with GBS colonisation (N=174)**	**Participants without GBS colonisation (N= 676)**	**p value***
Median age (IQR), years	25 (23-28) N=850	25 (23-28) N=174	25 (23-28) N=676	0.94
Age group, years				0.68
<20	69/850 (8.1%)	13/174 (7.5%)	56/676 (8.3%)	
20–<25	363/850 (42.7%)	79/174 (45.4%)	284/676 (42.0%)	
25–<30	313/850 (36.8%)	60/174 (34.5%)	253/676 (37.4%)	
30–<35	97/850 (11.4%)	19/174 (10.9%)	78/676 (11.5%)	
35–<40	8/850 (0.9%)	3/174 (1.7%)	5/676 (0.7%)	
≥40	0/850 (0.0%)	0/174 (0.0%)	0/676 (0.0%)	
Race or ethnicity				
Black	0/850 (0.0%)	0/174 (0.0%)	0/676 (0.0%)	
Asian	850/850 (100.0%)	174/174 (100.0%)	676/676 (100.0%)	
Other	0/850 (0.0%)	0/174 (0.0%)	0/676 (0.0%)	
Median gravidity (IQR)	1 (1-2) N=848	1 (1-2) N=174	1 (1-2) N=674	0.81
Gravidity				0.38
1	465/848 (54.8%)	97/174 (55.7%)	368/674 (54.6%)	
2	229/848 (27.0%)	40/174 (23.0%)	189/674 (28.0%)	
3	107/848 (12.6%)	24/174 (13.8%)	83/674 (12.3%)	
≥4	47/848 (5.5%)	13/174 (7.5%)	34/674 (5.0%)	
Median parity	1 (1-1) N=319	1 (1-1) N=65	1 (1-1) N=254	0.057
Parity				0.052
0	0/319 (0.0%)	0/65 (0.0%)	0/254 (0.0%)	
1	269/319 (84.3%)	50/65 (76.9%)	219/254 (86.2%)	
2	47/319 (14.7%)	13/65 (20.0%)	34/254 (13.4%)	
3	3/319 (0.9%)	2/65 (3.1%)	1/254 (0.4%)	
≥4	0/319 (0.0%)	0/65 (0.0%)	0/254 (0.0%)	
Previous stillbirth				0.37
Yes	17/228 (7.5%)	5/49 (10.2%)	12/179 (6.7%)	
No	211/228 (92.5%)	44/49 (89.8%)	167/179 (93.3%)	
Previous pregnancy loss				0.21
Yes	104/314 (33.1%)	15/59 (25.4%)	89/255 (34.9%)	
No	210/314 (66.9%)	44/59 (74.6%)	166/255 (65.1%)	
Median maternal MUAC (IQR)	29 (26-32) N=632	29 (26.5-32) N=139	28 (26-32) N=493	0.037
Chorioamnionitis				
Yes	0/850 (0.0%)	0/174 (0.0%)	0/676 (0.0%)	
No	850/850 (100.0%)	174/174 (100.0%)	676/676 (100.0%)	
Single or multiple birth				>0.99
Singleton	848/850 (99.8%)	174/174 (100.0%)	674/676 (99.7%)	
Twin	2/850 (0.2%)	0/174 (0.0%)	2/676 (0.3%)	

Antimicrobials in urine				0.18
Yes	56/769 (7.3%)	7/157 (4.5%)	49/612 (8.0%)	
No	713/769 (92.7%)	150/157 (95.5%)	563/612 (92.0%)	
Bacteriuria				<0.0001
Yes	38/841 (4.5%)	34/171 (19.9%)	4/670 (0.6%)	
No	803/841 (95.5%)	137/171 (80.1%)	666/670 (99.4%)	
Total number of births	850	174	676	
Mode of delivery				
Vaginal	850/850 (100.0%)	174/174 (100.0%)	676/676 (100.0%)	
Caesarean	0/850 (0.0%)	0/174 (0.0%)	0/676 (0.0%)	
Emergency	-	-	-	
Elective	-	-	-	
Median birthweight of neonate in kilograms (IQR)	3 (2.7-3.3) N=849	3 (2.7-3.3) N=174	3 (2.7-3.3) N=675	0.49

Data are n/N (%) unless otherwise specified. GBS=group B *Streptococcus*. MUAC=mid-upper arm circumference. Denominator is the overall number of participants (with available data) in the group identified in the column heading. For mode of delivery, the denominator is the total number of births. *p value comparing categorical variables using Fisher exact test or χ^2 test or comparing quantitative values using Wilcoxon rank sum test for the difference between the GBS positive and GBS negative groups.

Supplementary Table 4d: Demographics of the study population at Kenya at time of enrolment	**Overall (N = 801)**	**Participants with GBS colonisation (N=170)**	**Participants without GBS colonisation (N= 632)**	**p value***
Median age (IQR), years	25 (22-29) N=802	24 (22-29) N=170	25 (21-29) N=632	0.82
Age group, years				0.81
<20	155/802 (19.3%)	28/170 (16.5%)	127/632 (20.1%)	
20–<25	289/802 (36.0%)	68/170 (40.0%)	221/632 (35.0%)	
25–<30	213/802 (26.6%)	44/170 (25.9%)	169/632 (26.7%)	
30–<35	98/802 (12.2%)	20/170 (11.8%)	78/632 (12.3%)	
35–<40	38/802 (4.7%)	9/170 (5.3%)	29/632 (4.6%)	
≥40	9/802 (1.1%)	1/170 (0.6%)	8/632 (1.3%)	
Race or ethnicity				>0.99
Black	801/802 (99.9%)	170/170 (100.0%)	631/632 (99.8%)	
Asian	1/802 (0.1%)	0/170 (0.0%)	1/632 (0.2%)	
Other	0/802 (0.0%)	0/170 (0.0%)	0/632 (0.0%)	
Median gravidity (IQR)	2 (1-4) N=802	2 (1-3) N=170	2 (1-4) N=632	0.31
Gravidity				0.83
1	273/802 (34.0%)	62/170 (36.5%)	211/632 (33.4%)	
2	200/802 (24.9%)	43/170 (25.3%)	157/632 (24.8%)	
3	126/802 (15.7%)	26/170 (15.3%)	100/632 (15.8%)	
≥4	203/802 (25.3%)	39/170 (22.9%)	164/632 (25.9%)	
Median parity	2 (1-3) N=529	2 (1-3) N=108	2 (1-3) N=421	0.73
Parity				0.79
0	36/529 (6.8%)	8/108 (7.4%)	28/421 (6.7%)	
1	195/529 (36.9%)	39/108 (36.1%)	156/421 (37.1%)	
2	119/529 (22.5%)	26/108 (24.1%)	93/421 (22.1%)	
3	76/529 (14.4%)	18/108 (16.7%)	58/421 (13.8%)	
≥4	103/529 (19.5%)	17/108 (15.7%)	86/421 (20.4%)	
Previous stillbirth				0.13
Yes	25/529 (4.7%)	2/108 (1.9%)	23/421 (5.5%)	
No	504/529 (95.3%)	106/108 (98.1%)	398/421 (94.5%)	
Previous pregnancy loss				0.94
Yes	92/529 (17.4%)	18/108 (16.7%)	74/421 (17.6%)	
No	437/529 (82.6%)	90/108 (83.3%)	347/421 (82.4%)	
Median maternal MUAC (IQR)	25 (24-28) N=802	26 (24-28) N=170	25 (24-28) N=632	0.31
Chorioamnionitis				>0.99
Yes	1/802 (0.1%)	0/170 (0.0%)	1/632 (0.2%)	
No	801/802 (99.9%)	170/170 (100.0%)	631/632 (99.8%)	
Single or multiple birth				>0.99

Singleton	801/802 (99.9%)	170/170 (100.0%)	631/632 (99.8%)	
Twin	1/802 (0.1%)	0/170 (0.0%)	1/632 (0.2%)	
Antimicrobials in urine				0.79
Yes	21/777 (2.7%)	5/170 (2.9%)	16/607 (2.6%)	
No	756/777 (97.3%)	165/170 (97.1%)	591/607 (97.4%)	
Bacteriuria				<0.0001
Yes	72/802 (9.0%)	71/170 (41.8%)	1/632 (0.2%)	
No	730/802 (91.0%)	99/170 (58.2%)	631/632 (99.8%)	
Total number of births	803	170	633	
Mode of delivery				
Vaginal	716/803 (89.2%)	156/170 (91.8%)	560/633 (88.5%)	
Caesarean	87/803 (10.8%)	14/170 (8.2%)	73/633 (11.5%)	
Emergency	85/87 (97.7%)	13/14 (92.9%)	72/73 (98.6%)	0.3
Elective	2/87 (2.3%)	1/14 (7.1%)	1/73 (1.4%)	
Median birthweight of neonate in kilograms (IQR)	3.1 (2.8-3.4) N=802	3 (2.8-3.2) N=170	3.1 (2.8-3.4) N=632	0.12

Data are n/N (%) unless otherwise specified. GBS=group B *Streptococcus*. MUAC=mid-upper arm circumference. Denominator is the overall number of participants (with available data) in the group identified in the column heading. For mode of delivery, the denominator is the total number of births. *p value comparing categorical variables using Fisher exact test or χ^2 test or comparing quantitative values using Wilcoxon rank sum test for the difference between the GBS positive and GBS negative groups.

Supplementary Table 4e: Demographics of the study population at Mali at time of enrolment	**Overall (N = 764)**	**Participants with GBS colonisation (N=314)**	**Participants without GBS colonisation (N=450)**	**p value***
Median age (IQR), years	25 (21-30) N=764	25.5 (21-30) N=314	25 (20-30) N=450	0.12
Age group, years				0.16
<20	180/764 (23.6%)	65/314 (20.7%)	115/450 (25.6%)	
20–<25	234/764 (30.6%)	92/314 (29.3%)	142/450 (31.6%)	
25–<30	193/764 (25.3%)	90/314 (28.7%)	103/450 (22.9%)	
30–<35	92/764 (12.0%)	35/314 (11.1%)	57/450 (12.7%)	
35–<40	59/764 (7.7%)	28/314 (8.9%)	31/450 (6.9%)	
≥40	6/764 (0.8%)	4/314 (1.3%)	2/450 (0.4%)	
Race or ethnicity				
Black	764/764 (100.0%)	314/314 (100.0%)	450/450 (100.0%)	
Asian	0/764 (0.0%)	0/314 (0.0%)	0/450 (0.0%)	
Other	0/764 (0.0%)	0/314 (0.0%)	0/450 (0.0%)	
Median gravidity (IQR)	3 (2-5) N=764	3 (2-5) N=314	3 (2-5) N=450	0.22
Gravidity				0.62
1	155/764 (20.3%)	58/314 (18.5%)	97/450 (21.6%)	
2	170/764 (22.3%)	67/314 (21.3%)	103/450 (22.9%)	
3	124/764 (16.2%)	54/314 (17.2%)	70/450 (15.6%)	
≥4	315/764 (41.2%)	135/314 (43.0%)	180/450 (40.0%)	
Median parity	2 (1-4) N=609	2 (1-4) N=256	2 (1-4) N=353	0.48
Parity				0.92
0	17/609 (2.8%)	6/256 (2.3%)	11/353 (3.1%)	
1	172/609 (28.2%)	69/256 (27.0%)	103/353 (29.2%)	
2	126/609 (20.7%)	54/256 (21.1%)	72/353 (20.4%)	
3	96/609 (15.8%)	43/256 (16.8%)	53/353 (15.0%)	
≥4	198/609 (32.5%)	84/256 (32.8%)	114/353 (32.3%)	
Previous stillbirth				>0.99
Yes	45/609 (7.4%)	19/256 (7.4%)	26/353 (7.4%)	
No	564/609 (92.6%)	237/256 (92.6%)	327/353 (92.6%)	
Previous pregnancy loss				0.64
Yes	77/609 (12.6%)	30/256 (11.7%)	47/353 (13.3%)	
No	532/609 (87.4%)	226/256 (88.3%)	306/353 (86.7%)	
Median maternal MUAC (IQR)	29 (26-32) N=213	30 (26-32) N=84	29 (26-31) N=129	0.17
Chorioamnionitis				0.41
Yes	1/764 (0.1%)	1/314 (0.3%)	0/450 (0.0%)	
No	763/764 (99.9%)	313/314 (99.7%)	450/450 (100.0%)	
Single or multiple birth				0.2

Singleton	743/764 (97.3%)	302/314 (96.2%)	441/450 (98.0%)	
Twin	21/764 (2.7%)	12/314 (3.8%)	9/450 (2.0%)	
Antimicrobials in urine				0.44
Yes	41/762 (5.4%)	14/313 (4.5%)	27/449 (6.0%)	
No	721/762 (94.6%)	299/313 (95.5%)	422/449 (94.0%)	
Bacteriuria				
Yes	-	-	-	
No	-	-	-	
Total number of births	785	326	459	
Mode of delivery				
Vaginal	754/785 (96.1%)	310/326 (95.1%)	444/459 (96.7%)	
Caesarean	31/785 (3.9%)	16/326 (4.9%)	15/459 (3.3%)	
Emergency	31/31 (100.0%)	16/16 (100.0%)	15/15 (100.0%)	
Elective	0/31 (0.0%)	0/16 (0.0%)	0/15 (0.0%)	
Median birthweight of neonate in kilograms (IQR)	3 (2.7-3.4) N=779	3 (2.7-3.4) N=324	3 (2.7-3.4) N=455	0.35

Data are n/N (%) unless otherwise specified. GBS=group B *Streptococcus*. MUAC=mid-upper arm circumference. Denominator is the overall number of participants (with available data) in the group identified in the column heading. For mode of delivery, the denominator is the total number of births. *p value comparing categorical variables using Fisher exact test or χ^2 test or comparing quantitative values using Wilcoxon rank sum test for the difference between the GBS positive and GBS negative groups.

Supplementary Table 4f: Demographics of the study population at Mozambique at time of enrolment	**Overall (N = 785)**	**Participants with GBS colonisation (N=212)**	**Participants without GBS colonisation (N=573)**	**p value***
Median age (IQR), years	23 (20-28) N=785	24 (20.75-28.25) N=212	23 (20-28) N=573	0.19
Age group, years				0.23
<20	231/785 (29.4%)	53/212 (25.0%)	178/573 (31.1%)	
20–<25	273/785 (34.8%)	80/212 (37.7%)	193/573 (33.7%)	
25–<30	129/785 (16.4%)	40/212 (18.9%)	89/573 (15.5%)	
30–<35	80/785 (10.2%)	20/212 (9.4%)	60/573 (10.5%)	
35–<40	58/785 (7.4%)	18/212 (8.5%)	40/573 (7.0%)	
≥40	14/785 (1.8%)	1/212 (0.5%)	13/573 (2.3%)	
Race or ethnicity				>0.99
Black	784/785 (99.9%)	212/212 (100.0%)	572/573 (99.8%)	
Asian	0/785 (0.0%)	0/212 (0.0%)	0/573 (0.0%)	
Other	1/785 (0.1%)	0/212 (0.0%)	1/573 (0.2%)	
Median gravidity (IQR)	2 (1-4) N=784	2 (2-4) N=212	2 (1-4) N=572	0.35
Gravidity				0.81
1	204/784 (26.0%)	51/212 (24.1%)	153/572 (26.7%)	
2	217/784 (27.7%)	57/212 (26.9%)	160/572 (28.0%)	
3	135/784 (17.2%)	39/212 (18.4%)	96/572 (16.8%)	
≥4	228/784 (29.1%)	65/212 (30.7%)	163/572 (28.5%)	
Median parity	2 (1-3) N=580	2 (1-3) N=161	2 (1-3) N=419	0.48
Parity				0.54
0	60/580 (10.3%)	14/161 (8.7%)	46/419 (11.0%)	
1	203/580 (35.0%)	62/161 (38.5%)	141/419 (33.7%)	
2	131/580 (22.6%)	37/161 (23.0%)	94/419 (22.4%)	
3	87/580 (15.0%)	26/161 (16.1%)	61/419 (14.6%)	
≥4	99/580 (17.1%)	22/161 (13.7%)	77/419 (18.4%)	
Previous stillbirth				0.49
Yes	22/578 (3.8%)	8/160 (5.0%)	14/418 (3.3%)	
No	556/578 (96.2%)	152/160 (95.0%)	404/418 (96.7%)	
Previous pregnancy loss				0.0057
Yes	95/578 (16.4%)	38/161 (23.6%)	57/417 (13.7%)	
No	483/578 (83.6%)	123/161 (76.4%)	360/417 (86.3%)	
Median maternal MUAC (IQR)	27 (26-29) N=785	27 (25-29) N=212	27 (26-29) N=573	0.73
Chorioamnionitis				
Yes	0/785 (0.0%)	0/212 (0.0%)	0/573 (0.0%)	
No	785/785 (100.0%)	212/212 (100.0%)	573/573 (100.0%)	
Single or multiple birth				0.049

Singleton	779/785 (99.2%)	208/212 (98.1%)	571/573 (99.7%)	
Twin	6/785 (0.8%)	4/212 (1.9%)	2/573 (0.3%)	
Antimicrobials in urine				0.083
Yes	46/750 (6.1%)	7/205 (3.4%)	39/545 (7.2%)	
No	704/750 (93.9%)	198/205 (96.6%)	506/545 (92.8%)	
Bacteriuria				<0.0001
Yes	89/778 (11.4%)	84/211 (39.8%)	5/567 (0.9%)	
No	689/778 (88.6%)	127/211 (60.2%)	562/567 (99.1%)	
Total number of births	791	216	575	
Mode of delivery				
Vaginal	791/791 (100.0%)	216/216 (100.0%)	575/575 (100.0%)	
Caesarean	0/791 (0.0%)	0/216 (0.0%)	0/575 (0.0%)	
Emergency	-	-	-	
Elective	-	-	-	
Median birthweight of neonate in kilograms (IQR)	3.2 (2.9-3.4) N=791	3.2 (2.9-3.4) N=216	3.2 (2.9-3.4) N=575	0.65

Data are n/N (%) unless otherwise specified. GBS=group B *Streptococcus*. MUAC=mid-upper arm circumference. Denominator is the overall number of participants (with available data) in the group identified in the column heading. For mode of delivery, the denominator is the total number of births. *p value comparing categorical variables using Fisher exact test or χ^2 test or comparing quantitative values using Wilcoxon rank sum test for the difference between the GBS positive and GBS negative groups.

Supplementary Table 4g: Demographics of the study population at Nigeria at time of enrolment	**Overall (N = 874)**	**Participants with GBS colonisation (N=201)**	**Participants without GBS colonisation (N=673)**	**p value***
Median age (IQR), years	30 (27-34) N=874	31 (27-34) N=201	30 (27-34) N=673	0.35
Age group, years				0.35
<20	16/874 (1.8%)	3/201 (1.5%)	13/673 (1.9%)	
20–<25	117/874 (13.4%)	20/201 (10.0%)	97/673 (14.4%)	
25–<30	337/874 (38.6%)	77/201 (38.3%)	260/673 (38.6%)	
30–<35	266/874 (30.4%)	72/201 (35.8%)	194/673 (28.8%)	
35–<40	127/874 (14.5%)	26/201 (12.9%)	101/673 (15.0%)	
≥40	11/874 (1.3%)	3/201 (1.5%)	8/673 (1.2%)	
Race or ethnicity				
Black	872/872 (100.0%)	201/201 (100.0%)	671/671 (100.0%)	
Asian	0/872 (0.0%)	0/201 (0.0%)	0/671 (0.0%)	
Other	0/872 (0.0%)	0/201 (0.0%)	0/671 (0.0%)	
Median gravidity (IQR)	3 (2-4) N=871	3 (2-4) N=201	3 (2-4) N=670	0.36
Gravidity				0.41
1	185/871 (21.2%)	37/201 (18.4%)	148/670 (22.1%)	
2	217/871 (24.9%)	46/201 (22.9%)	171/670 (25.5%)	
3	189/871 (21.7%)	50/201 (24.9%)	139/670 (20.7%)	
≥4	280/871 (32.1%)	68/201 (33.8%)	212/670 (31.6%)	
Median parity	2 (1-3) N=624	2 (1-3) N=148	2 (1-3) N=476	0.71
Parity				0.57
0	52/624 (8.3%)	15/148 (10.1%)	37/476 (7.8%)	
1	239/624 (38.3%)	53/148 (35.8%)	186/476 (39.1%)	
2	162/624 (26.0%)	41/148 (27.7%)	121/476 (25.4%)	
3	108/624 (17.3%)	28/148 (18.9%)	80/476 (16.8%)	
≥4	63/624 (10.1%)	11/148 (7.4%)	52/476 (10.9%)	
Previous stillbirth				0.94
Yes	50/516 (9.7%)	11/121 (9.1%)	39/395 (9.9%)	
No	466/516 (90.3%)	110/121 (90.9%)	356/395 (90.1%)	
Previous pregnancy loss				0.91
Yes	239/617 (38.7%)	57/150 (38.0%)	182/467 (39.0%)	
No	378/617 (61.3%)	93/150 (62.0%)	285/467 (61.0%)	
Median maternal MUAC (IQR)	30 (28-32) N=872	30 (28-33) N=201	30 (28-32) N=671	0.04
Chorioamnionitis				
Yes	0/866 (0.0%)	0/199 (0.0%)	0/667 (0.0%)	
No	866/866 (100.0%)	199/199 (100.0%)	667/667 (100.0%)	
Single or multiple birth				0.72

Singleton	863/874 (98.7%)	198/201 (98.5%)	665/673 (98.8%)	
Twin	11/874 (1.3%)	3/201 (1.5%)	8/673 (1.2%)	
Antimicrobials in urine				0.085
Yes	67/805 (8.3%)	9/182 (4.9%)	58/623 (9.3%)	
No	738/805 (91.7%)	173/182 (95.1%)	565/623 (90.7%)	
Bacteriuria				<0.0001
Yes	82/874 (9.4%)	71/201 (35.3%)	11/673 (1.6%)	
No	792/874 (90.6%)	130/201 (64.7%)	662/673 (98.4%)	
Total number of births	885	204	681	
Mode of delivery				
Vaginal	600/885 (67.8%)	149/204 (73.0%)	451/681 (66.2%)	
Caesarean	285/885 (32.2%)	55/204 (27.0%)	230/681 (33.8%)	
Emergency	177/285 (62.1%)	33/55 (60.0%)	144/230 (62.6%)	0.84
Elective	108/285 (37.9%)	22/55 (40.0%)	86/230 (37.4%)	
Median birthweight of neonate in kilograms (IQR)	3.2 (2.9-3.5) N=884	3.2 (2.8-3.5) N=204	3.2 (2.9-3.5) N=680	0.26

Data are n/N (%) unless otherwise specified. GBS=group B *Streptococcus*. MUAC=mid-upper arm circumference. Denominator is the overall number of participants (with available data) in the group identified in the column heading. For mode of delivery, the denominator is the total number of births. *p value comparing categorical variables using Fisher exact test or χ^2 test or comparing quantitative values using Wilcoxon rank sum test for the difference between the GBS positive and GBS negative groups.

Supplementary Table 4h: Demographics of the study population at South Africa at time of enrolment	**Overall (N = 892)**	**Participants with GBS colonisation (N=275)**	**Participants without GBS colonisation (N=617)**	**p value***
Median age (IQR), years	25 (22-30) N=892	27 (22-32) N=275	25 (21-29) N=617	0.0051
Age group, years				0.035
<20	147/892 (16.5%)	39/275 (14.2%)	108/617 (17.5%)	
20–<25	306/892 (34.3%)	84/275 (30.5%)	222/617 (36.0%)	
25–<30	228/892 (25.6%)	68/275 (24.7%)	160/617 (25.9%)	
30–<35	128/892 (14.3%)	52/275 (18.9%)	76/617 (12.3%)	
35–<40	64/892 (7.2%)	23/275 (8.4%)	41/617 (6.6%)	
≥40	19/892 (2.1%)	9/275 (3.3%)	10/617 (1.6%)	
Race or ethnicity				0.92
Black	860/889 (96.7%)	265/274 (96.7%)	595/615 (96.7%)	
Asian	2/889 (0.2%)	0/274 (0.0%)	2/615 (0.3%)	
Other	27/889 (3.0%)	9/274 (3.3%)	18/615 (2.9%)	
Median gravidity (IQR)	2 (1-3) N=891	2 (1-3) N=275	2 (1-3) N=616	0.0044
Gravidity				0.031
1	357/891 (40.1%)	95/275 (34.5%)	262/616 (42.5%)	
2	277/891 (31.1%)	83/275 (30.2%)	194/616 (31.5%)	
3	163/891 (18.3%)	61/275 (22.2%)	102/616 (16.6%)	
≥4	94/891 (10.5%)	36/275 (13.1%)	58/616 (9.4%)	
Median parity	1 (1-2) N=534	1 (1-2) N=180	1 (1-2) N=354	0.16
Parity				0.6
0	76/534 (14.2%)	22/180 (12.2%)	54/354 (15.3%)	
1	254/534 (47.6%)	83/180 (46.1%)	171/354 (48.3%)	
2	145/534 (27.2%)	51/180 (28.3%)	94/354 (26.6%)	
3	39/534 (7.3%)	17/180 (9.4%)	22/354 (6.2%)	
≥4	20/534 (3.7%)	7/180 (3.9%)	13/354 (3.7%)	
Previous stillbirth				>0.99
Yes	20/522 (3.8%)	7/176 (4.0%)	13/346 (3.8%)	
No	502/522 (96.2%)	169/176 (96.0%)	333/346 (96.2%)	
Previous pregnancy loss				0.95
Yes	117/531 (22.0%)	40/178 (22.5%)	77/353 (21.8%)	
No	414/531 (78.0%)	138/178 (77.5%)	276/353 (78.2%)	
Median maternal MUAC (IQR)	29 (26-32) N=800	29 (27-32) N=244	28 (26-32) N=556	0.0017
Chorioamnionitis				0.71
Yes	8/886 (0.9%)	3/274 (1.1%)	5/612 (0.8%)	
No	878/886 (99.1%)	271/274 (98.9%)	607/612 (99.2%)	
Single or multiple birth				0.11

Singleton	885/892 (99.2%)	275/275 (100.0%)	610/617 (98.9%)	
Twin	7/892 (0.8%)	0/275 (0.0%)	7/617 (1.1%)	
Antimicrobials in urine				0.039
Yes	68/828 (8.2%)	13/256 (5.1%)	55/572 (9.6%)	
No	760/828 (91.8%)	243/256 (94.9%)	517/572 (90.4%)	
Bacteriuria				<0.0001
Yes	123/875 (14.1%)	114/271 (42.1%)	9/604 (1.5%)	
No	752/875 (85.9%)	157/271 (57.9%)	595/604 (98.5%)	
Total number of births	899	275	624	
Mode of delivery				
Vaginal	642/899 (71.4%)	192/275 (69.8%)	450/624 (72.1%)	
Caesarean	257/899 (28.6%)	83/275 (30.2%)	174/624 (27.9%)	
Emergency	246/257 (95.7%)	79/83 (95.2%)	167/174 (96.0%)	0.75
Elective	11/257 (4.3%)	4/83 (4.8%)	7/174 (4.0%)	
Median birthweight of neonate in kilograms (IQR)	3.2 (2.9-3.5) N=895	3.2 (2.9-3.5) N=273	3.2 (2.9-3.5) N=622	0.88

Data are n/N (%) unless otherwise specified. GBS=group B *Streptococcus*. MUAC=mid-upper arm circumference. Denominator is the overall number of participants (with available data) in the group identified in the column heading. For mode of delivery, the denominator is the total number of births. *p value comparing categorical variables using Fisher exact test or χ^2 test or comparing quantitative values using Wilcoxon rank sum test for the difference between the GBS positive and GBS negative groups.

Supplementary Table 5: Association between GBS colonization and demographic characteristics at enrolment.

Characteristics		Overall n (%)	Not colonised n (%)	Colonised n (%)	aOR (95% CI)	P value
Study site	South Africa	892 (13.7%) N=6514	617 (12.5%) N=4942	275 (17.5%) N=1572	Reference	Reference
	Bangladesh	788 (12.1%) N=6514	650 (13.2%) N=4942	138 (8.8%) N=1572	0.50 (0.39-0.64)	<0.0001
	Ethiopia	759 (11.7%) N=6514	671 (13.6%) N=4942	88 (5.6%) N=1572	0.31 (0.24-0.41)	<0.0001
	India	850 (13%) N=6514	676 (13.7%) N=4942	174 (11.1%) N=1572	0.59 (0.47-0.75)	<0.0001
	Kenya	802 (12.3%) N=6514	632 (12.8%) N=4942	170 (10.8%) N=1572	0.62 (0.49-0.78)	<0.0001
	Mali	764 (11.7%) N=6514	450 (9.1%) N=4942	314 (20%) N=1572	1.57 (1.26-1.96)	<0.0
	Mozambique	785 (12.1%) N=6514	573 (11.6%) N=4942	212 (13.5%) N=1572	0.86 (0.68-1.09)	0.21
	Nigeria	874 (13.4%) N=6514	673 (13.6%) N=4942	201 (12.8%) N=1572	0.62 (0.49-0.78)	<0.0001
Age	<20	1238 (19%) N=6512	969 (19.6%) N=4940	269 (17.1%) N=1572	Ref	Ref
	20 - <25	2148 (33%) N=6512	1642 (33.2%) N=4940	506 (32.2%) N=1572	1.15 (0.95-1.38)	0.15
	25 - <30	1835 (28.2%) N=6512	1399 (28.3%) N=4940	436 (27.7%) N=1572	1.15 (0.93-1.41)	0.21
	30 - <35	851 (13.1%) N=6512	617 (12.5%) N=4940	234 (14.9%) N=1572	1.28 (0.98-1.65)	0.07
	35 - <40	378 (5.8%) N=6512	269 (5.4%) N=4940	109 (6.9%) N=1572	1.26 (0.91-1.73)	0.16
	>=40	62 (1%) N=6512	44 (0.9%) N=4940	18 (1.1%) N=1572	1.41 (0.77-2.6)	0.27
Antimicrobials in the urine	No	5757 (92.5%) N=6227	4328 (91.7%) N=4721	1429 (94.9%) N=1506	Ref	Ref
	Yes	470 (7.5%) N=6227	393 (8.3%) N=4721	77 (5.1%) N=1506	0.64 (0.49-0.83)	0.00077
Gravida	1	2435 (37.5%) N=6485	1922 (39.1%) N=4913	513 (32.6%) N=1572	Ref	Ref

	2	1703 (26.3%) N=6485	1306 (26.6%) N=4913	397 (25.3%) N=1572	1.03 (0.87-1.21)	0.77
	3	1062 (16.4%) N=6485	769 (15.7%) N=4913	293 (18.6%) N=1572	1.17 (0.97-1.43)	0.11
	>3	1285 (19.8%) N=6485	916 (18.6%) N=4913	369 (23.5%) N=1572	1.05 (0.85-1.31)	0.63
Mode of delivery	NVD	5562 (85.5%) N=6509	4207 (85.2%) N=4938	1355 (86.3%) N=1571	Ref	Ref
	Emergency CS	828 (12.7%) N=6509	639 (12.9%) N=4938	189 (12%) N=1571	1.07 (0.88-1.3)	0.51
	Elective CS	119 (1.8%) N=6509	92 (1.9%) N=4938	27 (1.7%) N=1571	1.1 (0.68-1.79)	0.69

aOR: Adjusted Odd's Ratio

95% CI: 95% Confidence Interval

Supplementary Table 6: Frequency of Group B streptococcus colonization by a single or multiple serotypes.

	Colonized with GBS serotypes in women			Colonized with GBS serotypes in newborns			
Study Site	1	2	3	1	2	3	4
Bangladesh	90.6% (125/138)	8% (11/138)	1.4% (2/138)	96.3% (105/109)	3.7% (4/109)	0% (0/109)	0% (0/109)
Ethiopia	96.6% (85/88)	3.4% (3/88)	0% (0/88)	95.7% (89/93)	3.2% (3/93)	1.1% (1/93)	0% (0/93)
India	97.7% (170/174)	2.3% (4/174)	0% (0/174)	92% (149/162)	8% (13/162)	0% (0/162)	0% (0/162)
Kenya	94.7% (161/170)	5.3% (9/170)	0% (0/170)	91.6% (142/155)	7.7% (12/155)	0.6% (1/155)	0% (0/155)
Mali	75.8% (238/314)	20.1% (63/314)	4.1% (13/314)	72% (216/300)	22% (66/300)	5.3% (16/300)	0.7% (2/300)
Mozambique	91% (193/212)	9% (19/212)	0% (0/212)	92.1% (176/191)	7.9% (15/191)	0% (0/191)	0% (0/191)
Nigeria	91.5% (184/201)	8.5% (17/201)	0% (0/201)	93.1% (148/159)	5.7% (9/159)	1.3% (2/159)	0% (0/159)
South Africa	88.4% (243/275)	11.6% (32/275)	0% (0/275)	94.1% (222/236)	5.9% (14/236)	0% (0/236)	0% (0/236)
Overall	89.0% (1399/1572)	10.1% (158/1572)	1.0% (15/1572)	88.8% (1247/1405)	9.7% (136/1405)	1.4% (20/1405)	0.1% (2/1405)

Supplementary Table 7: Group B *Streptococcus* bacteriuria among mothers.

Site	GBS bacteriuria	Vertical transfer of Group B streptococcus to newborn at delivery.	Same serotype detected from Urine and vaginal/rectal swabs
Bangladesh	3.9% (2.8-5.5); [31/788]	93.5% (79.3-98.2) [29/31]	30/31 (96.8%)
India	4.5% (3.3-6.1); [38/841]	92.1% (79.2-97.3) [35/38]	31/34 (91.2%)
Kenya	9% (7.2-11.2); [72/802]	84.7% (74.7-91.2) [61/72]	68/71 (95.8%)
Mozambique	11.4% (9.4-13.9); [89/778]	91% (83.3-95.4) [81/89]	81/84 (96.4%)
Nigeria	9.4% (7.6-11.5); [82/874]	70.7% (60.1-79.5) [58/82]	62/71 (87.3%)
South Africa	14.1%(11.9-16.5); [123/875]	76.9% (68.6-83.5) [93/121]	109/114 (95.6%)
Overall	8.8% (8-9.6); [435/4958]	82.4% (78.6-85.7)[357/433]	381/405 (94.1%)

Supplementary Table 8: Frequencies and percent of missingness for each variable included in the multiple logistic regression model overall and by site.

Site	Maternal colonisation	Age	Antimicrobials in the urine	Gravida	Mode of delivery	Excluded from complete case analysis
South Africa	0 (0)	0 (0)	64 (7.2)	1 (0.1)	0 (0)	65 (7.3)
Bangladesh	0 (0)	1 (0.1)	0 (0)	0 (0)	0 (0)	1 (0.1)
Ethiopia	0 (0)	1 (0.1)	11 (1.4)	22 (2.9)	5 (0.7)	39 (5.1)
India	0 (0)	0 (0)	81 (9.5)	2 (0.2)	0 (0)	83 (9.8)
Kenya	0 (0)	0 (0)	25 (3.1)	0 (0)	0 (0)	25 (3.1)
Mali	0 (0)	0 (0)	2 (0.3)	0 (0)	0 (0)	2 (0.3)
Mozambique	0 (0)	0 (0)	35 (4.5)	1 (0.1)	0 (0)	36 (4.6)
Nigeria	0 (0)	0 (0)	69 (7.9)	3 (0.3)	0 (0)	72 (8.2)
Overall	0 (0)	2 (0)	287 (4.4)	29 (0.4)	5 (0.1)	323 (5)

References

1. Verani JR, McGee L, Schrag SJ. Prevention of perinatal group B streptococcal disease--revised guidelines from CDC, 2010. *MMWR Recommendations and reports : Morbidity and mortality weekly report Recommendations and reports* 2010; **59**(Rr-10): 1-36.

Study protocol

Group B *Streptococcus* colonization in mother-newborn dyads and association with serotype-specific capsular antibodies in low and middle income South Asian and African countries

Protocol Chair: Shabir A. Madhi

Sponsored by Bill and Melinda Gates Foundation (Grant number: OPP1117629)

Protocol Chair

- Shabir A. Madhi (ShabirM@nicd.ac.za)

MRC: Respiratory and Meningeal Pathogens Research Unit

DST/NRF Research Chair: Vaccine Preventable Diseases

1 Modderfontein Road, NHLS, Sandringham, South Africa

Tel: +27 11 386 6137

Study Co-investigators

- Clare L. Cutland (cutlandc@rmpru.co.za)
- Gaurav Kwatra (kwatrag@rmpru.co.za)
- Marta C. Nunes (nunesm@rmpru.co.za)

MRC: Respiratory and Meningeal Pathogens Research Unit

DST/NRF: Vaccine Preventable Diseases

Chris Hani Baragwanath Academic Hospital, Soweto, South Africa

Tel: +27 11 983 4283

Study Collaborators

- Stephanie Schrag (zha6@cdc.gov)

Epidemiology Team Lead, Respiratory Diseases Branch, NCIRD

Centers for Disease Control and Prevention, MS-C25

1600 Clifton Rd

Atlanta, GA 30333, USA

Tel: +1 404 639 4646

- Peter Adrian (adrianp@rmpru.co.za)

MRC: Respiratory and Meningeal Pathogens Research Unit

DST/NRF: Vaccine Preventable Diseases

Chris Hani Baragwanath Academic Hospital, Soweto, South Africa

Tel: +27 11 983 4283

Study Statisticians

- Alane Izu (izua@rmpru.co.za)

MRC: Respiratory and Meningeal Pathogens Research Unit

DST/NRF: Vaccine Preventable Diseases

Chris Hani Baragwanath Academic Hospital, Soweto, South Africa

Tel: +27 11 983 4283

- Nong Shang (nms6@cdc.gov)

Centers for Disease Control and Prevention, MS-C25

1600 Clifton Rd

Atlanta, GA 30333, USA

Study sites

Respiratory and Meningeal Pathogens Research Unit

Chris Hani-Baragwanath Academic Hospital

Chris Hani Road, Diepkloof

Gauteng, South Africa

Proposed study countries: Bangladesh, Bhutan, India, Ethiopia, Kenya, Mozambique, Nigeria, Pakistan**,** Philippines, Mali, South Africa

Contents

i. Abbreviations 6

1. Background 7

2. Group B *Streptococcus* colonization and disease 8

3. Aims and Objectives 14

4. Study framework 15

4.1 Study sites 15

4.2 Study population 16

4.3 Inclusion criteria 16

4.4 Exclusion criteria 16

5. Sample size 17

6. Participants enrolment and specimen collection 19

6.1 Specimen Collection 20

7. Laboratory Methods 21

7.1 GBS detection and isolation on-site 22

7.2 Vaginal swabs quality control at the central laboratory 22

7.3 Serotyping of GBS isolates at the central laboratory 22

7.4 Density of GBS colonization analysis at the central laboratory 23

7.5 Antibody analysis at the central laboratory 23

7.6 Training at the central laboratory 24

7.7 HIV testing 24

7.8 Malaria and Syphilis testing 25

8. Data Management 25

8.1 Data Processing 25

8.2 Data analysis 28

9. Protection of human research participants 31

10. Time frames for study conduct 33

11. Timelines 34

12. References 35

13. Appendix 1 37

14. Appendix 2 38

i. Abbreviations

GBS: Group B *Streptococcus*

EOD: Early onset disease

IAP: Intrapartum antibiotic prophylaxis

SOP: Standard operating procedure

RMPRU: Respiratory and Meningeal Pathogens Research Unit

QA: Quality assurance

ICF: Informed consent form

CAMP: Christie Atkins Munch-Petersen

QC: Quality control

qPCR: quantitative PCR

CPS: Capsular polysaccharide

1. Background

Despite the advances in reducing global under-5 mortality, including death due to pneumonia and diarrhea over the past two decades, there has been less progress in reducing mortality during the neonatal period. In 2013, it was estimated that 2,611,000 deaths (42%) of the 6,275,000 under-5 childhood deaths occurred during the first 28 days of life, including 2,001,400 which occurred within 7 days of life [1]. A major preventable cause of death during the neonatal period includes bacterial sepsis/meningitis (approximately 20% of all deaths). Group B *Streptococcus* (GBS) of which there are 10 serotypes of varying invasive potential is a major cause of invasive bacterial disease in neonates and young infants [2]. Furthermore, maternal infection by some bacteria, including chorioamnionitis due to GBS, may precipitate stillbirths and preterm labour. Complications due to premature birth are also leading causes of neonatal mortality globally.

Approximately two-thirds of invasive GBS disease occur during the first three days of life of which 75%-90% occur within 24 hours of birth in the absence of clinical interventions such as intrapartum antibiotic prophylaxis (IAP) [3]. A major challenge in delineating the causes of bacterial sepsis in low income countries, include limited diagnostic facilities in many settings for culture of blood or cerebrospinal fluid samples. This is further aggravated with many deliveries occurring outside of health care facilities, which coupled with a high proportion of babies dying within 24 hours of birth, results in many newborn deaths not being investigated for bacterial sepsis including in epidemiological studies. This could lead to a distortion in the role of different bacteria as causes of neonatal sepsis (especially in the newborn), with environmental pathogens being more likely to be identified in such studies. This is particularly pertinent for early-onset invasive GBS disease (EOD), which generally is diagnosed on blood samples collected within 24 hours of birth and not infrequently at the

time of birth, like was demonstrated in 75% of cases in South Africa and 90% of cases in USA prior to the IAP program to pregnant women colonized by GBS [3, 4]. Consequently an underestimation of the burden of EOD in resource-constrained countries would be reported. The relevance that access to health care following birth has on establishing the spectrum of bacteria associated with neonatal sepsis was illustrated in a population-based study from Bangladesh in which only 1 of the 30 confirmed cases of neonatal bacteraemia was due to GBS. However, of the 259 neonatal deaths, 62% were not investigated for bacteraemia, including 52% which occurred within 24 hours of birth [5]. It is therefore conceivable that most of the EOD cases in studies such as these are being missed and the contribution of GBS to neonatal mortality being significantly underestimated in settings where ill newborns are not timeously investigated for sepsis. This potential detection bias in neonatal sepsis epidemiological studies was recently corroborated in the neonatal sepsis aetiology study in South Asia (ANISA), where GBS was seldom identified. Notably, despite the intensity of community-based surveillance in ANISA, no blood samples were obtainable in 80% of the neonatal deaths, 70% of who died within 6 hrs of birth and 30% within the first hour of life (personal communication Samir Saha).

2. Group B *Streptococcus* colonization and disease

Invasive GBS disease in children <7 days age (i.e. early onset disease; EOD) results from vertical acquisition of GBS *in utero* by the foetus or during labour from recto-vaginally colonized women. The incidence of EOD in full-term newborns has declined by approximately 90% in countries where there is routine screening of pregnant women for GBS recto-vaginal colonization at 35-37 weeks of gestational age and where IAP is provided 4 hours prior to delivery to colonized women [6]. The strategy for routine screening for GBS colonization coupled with IAP, however, is not logistically feasible in

most resource-constrained countries and has also been shown to be not cost-effective including in some high-income countries [7].

Recto-vaginal GBS colonization of the mother at delivery has been identified as the major risk-factor for developing EOD [3]. It has been estimated that in the absence of IAP, approximately 40-50% of newborns born to GBS colonized women would be colonized at the time of birth, of whom 1-3% would develop EOD (Figure 1) [8]. These estimates were corroborated by a longitudinal cohort study of 5099 women in South Africa among whom the prevalence of vaginal colonization at delivery was 21%, with 57% of newborns to colonized women being colonized themselves at birth, and among whom the incidence of EOD was 2% [3, 9] (results of which are indicated by the black boxes in Figure 1).

Figure 1: Association between maternal GBS vaginal colonization, vertical GBS acquisition by the newborn and incidence of invasive GBS disease [3, 8, 9]

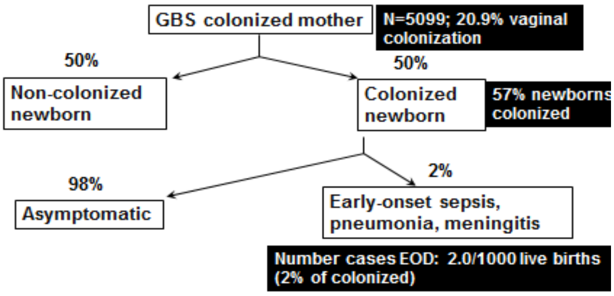

Supporting the association between prevalence of maternal GBS colonization and risk of developing EOD are data from USA prior to the era of IAP, where both the prevalence of colonization and incidence of EOD was greater in African-American compared to Caucasian women [10, 11]. These data suggest that the prevalence of GBS colonization in the mothers and vertical acquisition thereof by the newborns could serve as a crude proxy to estimate the incidence of EOD in newborns in settings where IAP is not standard-of-care.

Prevalence of GBS colonization in women at delivery across different world regions varies from 11.6% in South-East Asian to 21% in Africa (Table 1) [12]. Variability in sample

collection techniques and bacterial culture methods between regions might, however, contribute to the observed differences in colonization prevalence among regions.

Table 1: Meta-analysis on prevalence of maternal GBS colonization and incidence of early-onset disease [12, 13].

Region	Specimen Collection	Prevalence Range (%)	Prevalence Mean (%)	Incidence of EOD, per 1000 live births (95%CI)
Africa	Late pregnancy	16.5-28.4	22.5	0.53 (0.15–0.92)
	Delivery	20.8-21.0	20.9	
Americas	Late pregnancy	0.5-33.5	18.5	0.50 (0.43–0.57)
	Delivery	8.6-28.0	20.2	
Europe	Late pregnancy	10.6-37.9	20.0	0.45 (0.34–0.56)
	Delivery	8.0-29.3	18.7	
South-East Asia	Late pregnancy	2.3-16.0	10.3	0.11 (0.01–0.22)
	Delivery	8.6-18.1	11.6	

The current CDC recommendation for the isolation of GBS from vaginal and rectal or recto-vaginal swabs is by initial growth in a selective broth medium with antibiotics, followed by subculture on blood agar or selective media [14]. A literature review, from 1997 to 2014 on GBS colonization, identified only four studies from South Asia (which included three from India [15-17] and one from Bangladesh [18] (whilst no studies were identified from Pakistan and Bhutan) and four from Africa (including two from Zimbabwe, one from Malawi and one from South Africa [19-22]) that were conducted under the CDC recommended or equivalent guidelines. The current project will use standardized methods for sample collection and bacterial culture across different study sites, as used in a recent study in South Africa, in which the prevalence of recto-vaginal GBS colonization was 28% at ≥37 weeks of gestation age [20].

The incidence of EOD differs more markedly than the prevalence of GBS colonization among women at delivery between different regions [13]. A meta-analysis on EOD for the period 2000 to 2010, reported the highest incidence (per 1000 live births) in Africa (0.53, 95%CI: 0.15–0.92) and the lowest in South-East Asia (0.11, 95%CI: 0.01–0.22). Factors

which may contribute to this discrepancy might include geographic variability in: i) Prevalence of GBS colonization of pregnant women across regions and between countries, hence difference in exposure to the foetus/newborn; ii) Differences in density of GBS colonization among women, which could influence the rate of vertical acquisition by the foetus *in-utero* or newborn during birth; iii) Geographic differences in serotypes associated with colonization, with some colonizing serotypes (i.e. serotype III) being more invasive than others; iv) Differences in natural acquired protective serotype-specific capsular antibody levels in the women and/or among the newborns; and v) Differences in other risk-factors for EOD, such as prematurity rate, prolonged rupture of membranes, maternal GBS bacteriuria (also associated with density of colonization), maternal bacteriuria and exposure to any IAP.

It is possible that the reduced risk of developing EOD, despite similarity in exposure (i.e. maternal vaginal colonization) may be due to geographic differences in serotypes associated with colonization, with some serotypes being more invasive (Table 2) [23]. Although the invasive potential of GBS differs by serotypes, there are evidence that these are similar across different regions; with serotype III being the most invasive serotype globally (Table 2) [23]. Delineating the spectrum of serotypes (including relative prevalence of more virulent serotypes) across different settings, could contribute to determining whether heterogeneity in serotype distribution between regions may be contributing to the differences observed in EOD incidence. There is a paucity of data associated with maternal GBS colonization in low income countries, and particularly from South Asia.

Table 2: Estimation of invasive potential of GBS serotypes in different countries using serotype III as a referent serotype

Serotype	Country					
	**South Africa**	**Portugal [34]**	**Israel [36]**	**Sweden [31]**	**Netherlands [37]**	**Taiwan [37]**
Ia	163[a]/31[b] (0.49; 0.31–0.77)[c]	42/13 (1.52; 0.63–3.67)	12/10 (1.14; 0.41–3.18)	15/20 (1.37; 0.61–3.10)	24/6 (0.52; 0.63–3.67)	13/5 (0.22; 0.07–0.70)
Ib	36/7 (0.50; 0.22–1.18)	14/1 (0.35; 0.04–2.93)	9/2 (0.30; 0.05–1.57)	15/2 (0.14 0.03–0.64)	ND	5/2 (0.22; 0.04–1.27)
II	61/7 (0.30 0.13–0.67)	46/8 (0.85; 0.32–2.27)	23/14 (0.83; 0.34–2.03)	13/4 (0.32; 0.09–1.07)	ND	ND
III	202/78 (1.00)	59/12 (1.00)	26/19 1.00	36/35 (1.00)	20/33 (1.00)	19/34 (1.00)
IV	(20/5) (0.65; 0.23–1.79)	6/1 (0.81; 0.09–7.44)	ND	3/2 (0.69; 0.11–4.36)	ND	ND
V	55/8 (0.38; 0.17–0.83)	59/7 (0.58; 0.21–1.59)	18/9 (0.68; 0.25–1.85)	25/7 (0.29; 0.11–0.75)	14/4 (0.17; 0.05–0.60)	15/2 (0.07; 0.02–0.36)

In our data two isolates of the same serotype (i.e III) were obtained from the same infant, and only one isolate was included in the analysis.
[a]Value indicates colonizing isolates.
[b]Value indicates neonatal invasive isolates.
[c]Value in parenthesis indicates OR and 95% CI.
[d]ND: No data for either invasive, colonization or both isolates.
doi:10.1371/journal.pone.0017861.t003

The GBS isolates from previous studies in South Africa have been serotyped and their relationship to GBS sequence types and clonal complexes have been established. The South African data demonstrated that serotype/clonal complex combinations differ with respect to their invasive potential, and that the distribution of serotypes and clonal complexes are similar to those reported from Europe and the USA [23].

The observed disconnect between prevalence of maternal colonization and incidence of EOD in some regions may also be due to differences in serotype-specific capsular polysaccharide (CPS) antibody concentration of maternal origin in the newborns. An association between high maternal and newborn serotype-specific CPS antibody levels and reduced risk of EOD in newborns born to GBS colonized women has been demonstrated in studies from high income countries [24-28]. Cross-sectional studies have also identified significantly higher concentrations of serotype-specific CPS antibodies in women colonized by the homotypic serotype than in non-colonized women. These studies, have differed in serotype III–specific antibody concentration in relation to colonization status, suggesting that maternal antibody response to serotype III, the most important serotype associated with invasive GBS disease, may vary between regions, possibly because of differences in circulating strains or in host response [10, 29]. Limitations of these early serological studies

include differences in the serological assay methods and lack of standardized reference serum. This may also contribute to the differences in threshold of serum CPS antibody proposed as correlate of protection against invasive GBS disease. These gaps are currently being addressed by the Respiratory and Meningeal Pathogens Research Unit (RMPRU) in collaboration with Novartis (the leader in the field of developing a GBS conjugate vaccine).

To measure serotype-specific CPS IgG in serum we have recently established a Luminex multiplex IgG assay in our Unit. Similar to previous studies, we showed that recto-vaginal colonization with serotype Ia, III or V at birth was associated with higher serum CPS IgG to the homotypic colonizing serotypes (Clinical Microbiology and Infection, in press). However, in our longitudinal cohort study, new GBS serotype acquisitions (Ia, III and V) between 20 to 37 weeks of gestational age were inversely correlated with serotype-specific CPS serum IgG levels and opsonophagocytic activity at 20 weeks of gestational age (Clinical Microbiology and Infection, in press).

The aim of this proposal is to use standardised methodologies across at least eight low income Asian and African countries, to investigate for the prevalence of GBS vaginal colonization at the time of labour in the women and vertical transmission to their newborns. This will be coupled to evaluating maternal and newborn capsular antibody levels to specific GBS serotypes, to investigate for geographic variability in natural acquired CPS antibody and transplacental transfer ratio to their newborns. The mother-newborn colonization and capsular antibody levels will be sued to model the anticipated incidence of EOD in the various study settings.

3. Aims and Objectives

This study aims to inform the epidemiology of GBS in low-middle income settings. The study will address some of the critical limitations of the current data from low-middle income countries by using standardized methodologies for evaluating colonization and natural acquired serum CPS antibodies. The specific objectives of the study are:

<u>Primary Objective</u>

i) Define the prevalence of GBS colonization in HIV-uninfected pregnant women at term-delivery in low-middle income settings in Africa and South Asia.

ii) Determine serotype specific capsular antibody levels in HIV-uninfected pregnant women at term-delivery in low-middle income settings in Africa and South Asia.

<u>Secondary Objectives</u>

Colonization:

i) Determine the vertical transmission of GBS to the newborns in low-middle income settings in Africa and South Asia.

ii) Determine the serotypes associated with GBS colonization in pregnant women.

iii) Evaluation of the density of GBS colonization among pregnant women in low-middle income settings in Africa and South Asia.

Serology:

iv) Evaluate natural acquired maternal and cord-blood serotype-specific (serotypes Ia, Ib, III and V) capsular antibody levels at birth, and ratio of transplacental antibody transfer.

Modelling for EOD incidence:

v) Model the expected rates of EOD in the different settings using the measured variables related to GBS colonization and CPS antibody, together with other population based risk-factor measures (such as premature rate, IAP exposure, prolonged rupture of membranes, maternal HIV seroprevalence and maternal malaria infection rates).

Whilst the results of the study will address some of the key factors which might influence susceptibility to developing EOD, it is not designed to specifically measure the incidence of invasive GBS disease. Nevertheless, the present approach will clarify some of the postulated reasons which have been offered (in the absence of compelling evidence) for the differences observed in incidence of invasive GBS disease in low income settings. The data derived from the current project, coupled with population based data on other factors associated with risk for developing invasive GBS disease in newborns will be used to model the anticipated rates of EOD in the different countries.

4. Study framework

4.1 Study sites

This study is planned to be undertaken in at least 9 low middle income countries. The targeted countries include Bangladesh, Bhutan, India, Ethiopia, Kenya, Mozambique, Nigeria, Pakistan**,** Philippines, Mali and South Africa and a final decision as to their inclusion will be undertaken following site evaluation to ensure site capacity and capabilities to undertake this study. This evaluation will be led by investigators from RMPRU (in the capacity of Protocol Chair and grant awardee).

4.2 Study population

This study will be focused on pregnant women and their newborns who are delivered at term (≥37 weeks of gestation age) by normal vaginal delivery. Gestational age staging will be determined by extrapolation of the date of the last menstrual period and corroborated by physical examination of mother and/or infant and/or ultrasound report if available.

Informed consent will be obtained from the women on their behalf and of their infants either at the time of attending antenatal clinic visits or during the early stages of labour. The consenting process will be customized at the discretion of the study site.

4.3 Inclusion criteria

(i) Pregnant women age 18 to 45 years age.

(ii) Gestational age (GA) at antenatal consenting >34 weeks, or GA at delivery ≥37 weeks of gestation documented by the approximate date of the last menstrual period and corroborated by physical exam, or ultrasound examination if available.

(iii) Able to understand and comply with planned study procedures.

(iv) Provides written informed consent prior to initiation of study.

(v) Documented to be HIV-uninfected prior to study-enrolment (with testing of HIV only being necessary in countries where the background prevalence of HIV in pregnant women is >1%).

4.4 Exclusion criteria

(i) Unwilling to consent to study inclusion.

(ii) Antibiotics treatment in the two weeks prior to delivery.

(iii) Unable to obtain maternal blood.

(iv) Blood transfusion in the 30 days before delivery.

5. Sample size

The study sample size has been powered for the co-primary objective of the study, i.e. to determine GBS vaginal colonization prevalence in pregnant women at delivery at the different study sites. A sample size of 772 is required to detect at least 15% prevalence of colonization and 100 colonization events with ±5% precision (Table 3).

Table 3: Sample size to detect 10%, 15%, 20% and 25% maternal GBS colonization prevalence

Estimated maternal GBS colonization prevalence	Sample size
10%	1161
**15%**	**772**
20%	576
25%	459

Assuming a low colonization prevalence of 15% (n=116), the study will have 80% power to detect a 30% difference in the proportion of vertical GBS transmission to newborns compared to an anticipated 50% vertical transmission with α=0.05 (Table 4). 780 mothers will be target for enrolment at each site.

Table 4: Sample size to detect 10%, 15%, 20%, 25% and 30% difference in vertical transfer of GBS colonization, relative to a referent of 50% expected transmission

Estimated difference in GBS colonization in newborns	Sample size
10%	1605
**15%**	**719**
20%	408
25%	262
30%	103

To evaluate the serotype-specific CPS antibody levels in the mothers at the time of delivery we based the sample size on the South African data with 80% power and α=0.05. Taking in consideration that for serotype Ia, III and V an anticipated 46.3%, 35.3% and 64.7%, respectively, of pregnant women in South Africa at ≥37 weeks gestation (irrespective of colonization status) have antibody titer ≥0.5 µg/ml we estimated that a sample size of 297, 455 and 158 is required for serotype Ia, III and V, respectively to detect at least a 25% difference in the proportion of pregnant women with antibody titers ≥0.5 µg/ml compared to women in South Africa (Table 5). The sample size is also adequate to detect at least a 25% difference in the proportion of mothers with antibody titers ≥1.0 µg/ml for serotypes Ia and V compared to pregnant women with antibody titer >1 µg/ ml for serotype Ia and V in South Africa (Table 6).

Table 5: Prevalence of serotype-specific antibodies in women at ≥37 weeks of gestational age in South Africa (unpublished data)

**Antibody titer**	**Proportion of pregnant women with serotype specific antibody titers**		
	**Ia**	**III**	**V**
≥0.5 µg/ml	46.3%	35.3%	64.7%
≥1.0 µg/ml	39.0%	21.6%	40.2%
≥2.0 µg/ml	31.1%	14.9%	20.5%

Table 6: Sample size calculation to detect 25% difference in proportion of mothers with antibody above specified threshold

**Antibody titer**	**Sample size to detect 25% difference**		
	**Ia**	**III**	**V**
>0.5 µg/ml	297	455	158
>1.0 µg/ml	395	861	377
>2.0 µg/ml	536	1354	927

6. Participants enrolment and specimen collection

Participant recruitment (n=936) and sample collection will be conducted by trained study staff at the local community antenatal clinics or hospitals. Pregnant women will be approached for enrolment of themselves and their newborns into the study either at the time of attending antenatal clinics, during the early stages of labour or immediately following delivery and informed about the nature of the study. Interested women will be screened for eligibility criteria and given informed consent forms to read. Consenting and eligible women will be allocated a unique identification number, demographic and pregnancy related data will be collected. Specimens will be collected from the mother and the newborn at the time of delivery.

A 28-day after birth follow-up telephonic or in-person contact will be attempted for all infants enrolled. During this call the health status of infants will be reviewed with emphasis on sepsis and possible invasive disease.

The study staff at the different study sites will be trained using standard operating procedures (SOPs) developed at RMPRU, South Africa, on how samples should be collected. A phased-in approach will be undertaken with regard to site-initiation, with one site each initiated in Asia and one site in Africa, in the first phase of the project. These sites will target completion of enrolment within 9 months, with evaluation of the site's laboratory and clinical facility undertaken at the time of site initiation. Included in the site assessment will be quality evaluation at the central laboratory (RMPRU) for GBS isolation. The second phase of site initiations will 6 months following initiation of the initial sites, and will be staggered over a 12-month period to enable adequate oversight by the Core team prior and at the time of site initiation. The total study enrolment period across the sites, using this phased-in approach is estimated to occur over a period of 24 months.

6.1 Participant enrolment in South Africa

Participant recruitment, sample collection and follow up will be conducted by study staff of RMPRU at the Lillian Ngoyi local community clinic and Chris Hani Baragwanth Academic Hospital (CHBAH). The study staff will be trained using standard operating procedures available at RMPRU on sample collection and processing.

6.2 Specimen Collection

Swab for GBS culture: Separate lower vaginal and rectal swabs will be collected from the mothers at delivery, prior rupture of membranes where possible; and separate skin (surface swabs of the umbilicus, outer ear and axillary fold), rectal and throat swabs from the baby prior to washing of the newborn will be collected for GBS culture. Swab samples will be collected with rayon-tipped swabs that will be placed into separate Amies transport medium without charcoal. Swabs will be transported on ice to the site laboratory and processed within 24 hours of collection for GBS detection and isolation. However, if processing of swab is delayed, it will be stored at 4°C and will be processed within 72 hours of collection. A detailed SOP on the methods for swab collection and processing of the samples will be provided.

Swab for quality control and qPCR: Duplicate vaginal swabs from all mothers will be collected for evaluating density of GBS colonization by quantitative PCR (qPCR) and for quality control (QC) at the central laboratory in South Africa. The duplicate swabs will be collected into Amies transport medium and once at the local laboratory will be stored in STGG media at -70°C. All these swabs will be shipped on dry ice to the central laboratory (RMPRU). Duplicate vaginal swabs collected in South Africa will be directly processed in the central laboratory (RMPRU).

Maternal and cord blood: Maternal blood at the time of delivery and cord blood samples will be collected from each pair. After whole blood centrifugation serum with no further processing will be stored at -70ºC and shipped on dry ice to the central laboratory in South Africa (RMPRU) for antibody analysis. Blood samples collected in South Africa will be directly processed in the central laboratory (RMPRU).

Maternal urine sample: Maternal urine sample before delivery will be collected for GBS culture. All Urine samples will be stored at -70° C at the local facility and will be shipped on dry ice to central laboratory in South Africa (RMPRU) for antimicrobial testing. Urine collected in South Africa will be directly processed in the central laboratory (RMPRU) for GBS culture and antimicrobial testing.

See flow chart of trial procedures - Appendix 1.

7. Laboratory Methods

The justification for the centralization of testing of samples relates to the need to ensure quality and consistency of the testing and of the results obtained.

At the moment there is no standardised assay for antibody-quantification, so needing to use single platform which the central Unit has established and that is being validated against a Novartis assay. Also experience with GBS serotyping is limited at most facilities, and this project will not able to invest in training for this component which will not be sustainable and rarely used on a frequent basis.

7.1 GBS detection and isolation on-site

Once samples arrive at the local laboratory swabs will be inoculated onto selective media (CHROMagar StrepB; CA) and into selective broth (Todd-Hewitt broth supplemented with colistin and nalidixic acid; Lim broth). The selective broth will be further subcultured on selective media [14]. GBS-like colonies will be isolated and confirmed as GBS by testing for Christie Atkins Munch-Petersen (CAMP) factor, inability to hydrolyze esculin, catalase negativity and group B antigen detection. All positive GBS isolates will be stored in STGG medium at -70°C and shipped on dry ice to the central laboratory. A thorough quality assurance (QA) program between the sites will be developed (Appendix 2 for details) so that swabs will be analysed using standardized culture methods across all the sites. To ensure that identical methodology is used between sites, SOPs have been developed for: i) sample collection; ii) GBS culture (including details on culture media); iii) storage of isolates; and iv) storage of the specimens.

7.2 Vaginal swabs quality control at the central laboratory

The duplicate maternal vaginal swab stored in STGG media will be sent to the central laboratory in South Africa. 10% of these specimens will be re-tested for the presence of GBS using the detection method described above.

7.3 Serotyping of GBS isolates at the central laboratory

Once the GBS isolates are received in South Africa they will be serotyped using latex agglutination assay with specific antisera against types Ia, Ib and II to IX CPS antigens (Statens Serum Institute, SSI, Sweden). GBS isolates that tested negative by latex agglutination for all serotypes tested will be further molecular typed by PCR using serotype-specific primer sequences as described [30]. Furthermore, GBS isolates will be

archived in STGG media at -70°C for future strain characterization by multilocus sequence typing. The GBS isolation, serotyping, and strain typing techniques have been established at the RMPRU laboratory [20, 23].

7.4 Density of GBS colonization analysis at the central laboratory

The duplicate maternal vaginal swabs stored in STGG media will be sent to the central laboratory in South Africa. Evaluation of the density of GBS colonization among pregnant women will be done by real-time PCR on these specimens. Briefly, storage medium in which maternal duplicate vaginal samples were submerged will be used for DNA extraction using the easymag platform (BioMerieux) according to the manufacturer's instructions. GBS nucleic acid detection by real-time PCR will either be based on targeting the *cfb* gene which encodes the CAMP factor or the *sip* gene which encodes the surface immunogenic protein (Sip). Serial dilutions of known GBS type strain DNA will be used to produce a standard curve. Positive controls (purified DNA of known concentration) and negative controls (purified water) will be included in each run.

7.5 Antibody analysis at the central laboratory

For serotype-specific CPS antibody detection maternal and cord blood samples will be collected at each site and serum will be stored at -70°C and shipped on dry ice to the central laboratory. Detection of CPS antibodies in the serum samples will be done using multiplex Luminex assay for serotypes Ia, Ib, III and V at the central laboratory at RMPRU. The Unit uses a validated quantitative multiplex Luminex assay to determine antibody titers. Capsular polysaccharide antigens will be sourced from our collaborators at Novartis Vaccines (or other source), and the antibody titers will be measured against an in-house pooled reference serum that has been bridged with calibrated reference sera from our academic collaborators. All the

serum samples will be archived at -70°C for future analysis, in the event that global accepted standardized assay becomes available.

7.6 Training at the central laboratory

Laboratory technologists from the different participating sites will undergo laboratory training (approximately for 10 days) just prior the site starts recruitment on the standardized methods of GBS culture and identification. This will include theoretical training on laboratory SOPs and practical laboratory training on the techniques referred above, and will be conducted in accordance with the central laboratory training SOPs. The training will be performed in phases to ensure that attendees from each study site get close supervision, and that the knowledge gained is recent with respect to the initiation of the study.

External quality assessment (EQA) will be performed prior to the initiation of clinical samples collection on each site (Appendix 2). To ensure consistency of laboratory processing across all study sites, all laboratory consumables will be supplied by the central laboratory, of particular importance is the selective enrichment broth and Chromagar selective media.

7.7 HIV testing

Study sites with background prevalence of HIV in pregnant women >1% will required an HIV testing on the mother before consenting. This HIV test used will be the routine test used in each particular country and will be undertaken following informed pre- and post-test counselling. For countries where this is not undertaken as part of standard of care and where it is required, the testing will be undertaken through this study protocol. The management of women identified as being HIV-infected in this study will be based on the local standard of care for HIV-infected individuals and they will not be included in this

study, to reduce the confounding effect that maternal HIV-infection could have on maternal colonization and antibody levels.

7.8 Malaria and Syphilis testing

In malaria endemic countries malaria testing will be undertaken as part of standard of care. A test for syphilis will be required, this syphilis test used will be the routine test used in each particular country and will be generally undertaken as part of standard of care. For countries where this is not undertaken as part of standard of care, the testing will be undertaken through this study protocol.

8. Data Management

8.1Variables to be collected

The following data variables will be collected/ recorded for study participants when available:

a. Maternal age: Date of birth, date of enrolment
b. Gestational age (GA): Date of last normal menstrual period, symphysis-fundal height & date, sonar date and details if available. GA at delivery will be extrapolated.
c. Informed consent procedure: Date, language, how many people signed and who, questions asked, Witness
d. HIV uninfected- confirmation
e. Antibiotics in previous 2 weeks
f. Race: black, white, Asian

g. Marital status, Smoker, substance abuse

h. Practices which may affect colonization: vaginal douching

i. Housing, diet, livestock (cattle)

j. Occupation

k. Parity, gravidity, number of previous live births, IUFD/ stillbirths (GA-months x/9), abortions (induced/ spontaneous). Month & year of previous deliveries (pregnancy spacing)

l. Co-morbidities: hypertension, diabetes mellitus, malaria (most recent episode-month & year) STD: syphilis

m. Location of delivery- hospital/ clinic/ home

n. Birth attendant (categories- doctor, nurse, trained birth attendant, family member)

o. Rupture of membranes: date, time (duration calculated by database), not recorded

p. Delivery-related complications: APH, PPH

q. Evidence of infection in mother: Maternal fever (>38.0°C)- pre/ post delivery, tachycardia, chorioamnionitis recorded in chart, UTI, unexpected uterine tenderness, PVD (Yes/ no)

r. Evidence of foetal distress: MSL, foetal tachycardia, abnormal CGT (Yes/ no/ not available)

s. Recent concomitant medications: 2 weeks prior to and during L&D, Antibiotics, anti-malarials, blood products, Ig

t. Number of infants (CRF per infant)

u. Mode of delivery: Vaginal- cephalic/ breech/ assisted cephalic, Caesarian-emergency/ elective

v. Gender, birth weight (grams), APGAR score

w. Date and time of birth

x. Birth outcome: Live/ stillbirth/ admitted to neonatal unit

y. Details of samples collected

8.2. Data Processing, management and storage

All data collected from participants will be stripped of any identifiers that reveal the identity of that individual (beyond the use of participant ID). Screening logs will be maintained at the study site. Data will be collected on study-specific data collection forms, and entered into study-specific secure online databases at each study site, using password-protected computers. Collection forms and databases will be uniform across sites and a complete database will be compiled by the central Unit in South Africa. Paper records will be stored safely in locked cabinets at local investigational site for the duration of the study, and archived at the end of the study. Access to paper and electronic records will be restricted to authorised individuals.

Data management will be co-ordinated centrally at RMPRU using a secure online Electronic Data Capture system which provides intuitive interface for validated data entry; audit trails for tracking data manipulation and export procedures; automated export procedures for seamless data downloads to common statistical packages; and procedures for importing data from external sources). Internal monitoring and quality assurance will be conducted on important variables and the inform consent forms at each site by the local data teams. The database will include internal validity checks and ranges. On a regular basis the study local data manager, will generate a set of data listings that will be reviewed by the principal investigator at the site and key members of the team.

Sample collection and processing results will also be entered onto this database. The central team in South Africa will be able to review daily recruitment, sample collection, data uploads and sample shipping details.

8.3 Data analysis

The planned strategy is an attempt to delineate if any of the proposed factors (prevalence of vaginal colonization at birth, serotype distribution of colonizing isolates and serotype-specific CPS antibodies in the mothers and transfer thereof to their newborns) might be contributing to the differences observed in the burden of GBS EOD across the world. These data will also be interpreted in the context of prevalence of other obstetrical practices at the sites, e.g. utilization of IAP (risk-based or not), intrapartum fever, caesarean section rates, number of births outside of health care center and practices for investigating for sepsis.

The statistical plan of analysis to approach the primary and secondary objectives of the study will be developed. To model the EOD incidence the statistical analyses plan involves three steps of analyses. The first step answers the first question above, namely to build relationships between the proposed factors and the GBS EOD status. The next two steps attempt to generalize the findings outside of the study sites, or to answer the second question above. Details of the three steps of analyses are:

i) Apply semi-supervised learning methods to establish relationships between the proposed factors (prevalence of vaginal colonization at birth, serotype distribution of colonizing isolates and serotype-specific CPS antibodies in the mothers and transfer thereof to their newborns) and GBS EOD. Data collected from this study, which is mostly unlabelled in terms of the EOD status, will be combined with a

labelled training data from previous study performed in South Africa in order to generate prediction rules that are more accurate than using either of the two data sets alone. Data available from South Africa include: incidence of EOD, maternal prevalence of GBS colonization, vertical transmission to newborns, GBS serotypes associated with colonization and with invasive disease, proportion of community controls with serotype-specific CPS antibodies, prevalence other risk factors (e.g. premature birth, prolonged rupture of membranes, bacteruria) and utilization of IAP. The ideal product is a partitioning of the data space such that the individual participants within each subspace would share similar risk in developing EOD. The analysis might be expanded to a multi-level analysis with population level factors added to the four factors to improve the prediction accuracy.

ii) Parametric models, such as binary logistic regression or multinomial logistic regression, will be developed to build relationships between the population level factors with the partitioning of participants from step 1 above, either individually within each subspace or jointly for all subspaces. Bayesian methods will be applied with prior knowledge of the population level factors using a Polya-Gamma latent variable technique.

iii) Extrapolate the (posterior) parametric models from step 2 above to any specific area outside of the study sites of this proposed study to estimate the burden of EOD in the specific area.

The reasoning behind the proposed statistical analyses is that although the study will not directly measure the burden of EOD or late-onset disease (LOD), the collected data will be useful in simulating the expected incidence of disease, by measuring the prevalence of

maternal GBS colonization, vertical acquisition by the newborns and adjusting for any differences that may exist in serotype exposure and maternal capsular antibody levels.

In addition to the above proposed statistical analysis, descriptive summary statistics comparing maternal and infant responses and the relationships will be summarized by correlations analysis. Geometric mean concentrations (GMC; 95% confidence intervals), medians (inter quartile ranges) will be compared by cross tabulations using parametric or non-parametric tests as appropriate. For categorical variables, groups will be compared with either chi-square test or Fisher's two tailed exact test as appropriate. Antibody responses will be suitably log-transformed to satisfy the normality for the analyses and serotype-specific CPS antibody GMCs in the mother and newborn will be calculated; proportion of participants with antibody levels above pre-specified thresholds, which are proposed putative measures of protection against EOD in the newborns, will be determined. Analysis of factors which may affect transplacental antibody transfer ratio between the sites will be undertaken.

8.4. Intellectual Property

Data derived from the multicentre project will be held in the custodianship of the RMPRU. Individual investigators will share custodianship of their site-specific data sets with RMPRU. After publication of the main papers, the pooled anonymised dataset will be made available to other researchers, and access managed by the data management unit, and overseen by the RMPRU study team. Site-specific data will be managed by local data managers. The anonymity of all study participants will be ensured at all times. Future research on data collected must first be approved by a national independent expert committee, the overall principal investigator (S.A.Madhi) and the international scientific

advisory committee (which oversees all the study sites allied to GBS-MCS to ensure participants' safety and rights are respected).

9. Protection of human research participants

Confidentiality: Participants will be identified for study purposes with a unique numerical identifier. All laboratory specimens, evaluation forms, reports, and other records will be identified only by the numerical identifier to maintain participant confidentiality. All records will be kept in a secured area. All computer entry and networking programs will be done with coded numbers only. Participant identifiable information will not be published. All data collection activities will be carried out with the normal respect towards privacy, dignity and confidentiality.

Review board approval: The protocol and consent forms at the study sites will be approved by the local Human Research Ethics Committee (HREC) prior to initiation of the study.

Risks:

Sampling procedures that involve collection of maternal and cord blood, maternal urine samples, vaginal and rectal swabs and infant swabs (skin, rectal and throat) will be carefully explained to the participants in their own language. All procedures will be explained to mothers and undertaken by appropriately trained staff. Any participant found to have any medical problem such a positive urine culture will be referred to the appropriate and available health care provider at site. Risks relating to confidentiality regarding samples and data collected is guided by an international ethical framework and adherence to the regulations of independent national ethical bodies. Individual names are removed from all samples and data collected, and replaced by codes (participant identifier), to ensure that samples and data can

only be linked to the participants by people closely concerned with the research. Future research on samples and data collected must first be approved by a national independent expert committee, and the Human Research Ethics Committee, University of Witwatersrand, Johannesburg, South Africa, that oversees all the study sites allied to GBS-MCS to ensure participants' safety and rights are respected.

There is a small risk of bacterial infection at the venepuncture site associated with collection of blood specimens. To minimize this risk, sterile techniques will be employed.

Anticipated benefits: There are no direct benefits to enrolled participants; except that participation in the study will contribute further to medical knowledge.

Informed consent: Detailed written information about the study will be provided to potential participants by trained study staff. Fieldworkers and other study staff will be freely available to study participants throughout the study duration to answer any questions. Consent forms will be translated into local languages and contingency made for illiteracy. Study participants will know they can withdraw from the study at any time without having to give a reason.

Costs to participants: Participants will incur no extra costs based on participation in the study. No additional study-specific visits are required, as all the study procedures will be completed while they are in the labour ward. Participants will also be reimbursed R100 transport cost for participating in the study.

Disseminating results to the public: Summary results will be reported to the HREC and local IRBs at least bi-annually and on completion of the study. Community representatives, local health care providers and relevant government representatives will be updated regularly by local investigators. Results may be presented at professional clinical meetings and

national or international scientific meetings. Results will be submitted for publication in a peer-reviewed journal, where they will also be available to the public.

10. Time frames for study conduct

HREC submission date: 6th February 2015

Recruitment of participants: First two study sites will start recruitment in April 2015. Last non-South African study sites will conclude recruitment in third quarter of 2017

Participant recruitment at South African site will be initiated in August 2017, once approval for protocol amendment has been received. Recruitment is expected to last 6 months.

Funding: Bill and Melinda Gates Foundation (Grant number: OPP1117629)

11. Timelines

	2014		2015											
	Nov	Dec	Jan	Feb	Mar	Apr	May	Jun	Jul	Aug	Sep	Oct	Nov	Dec
Protocol development/site selection/site application, with facilities description and draft budget	■	■												
Site IRB submission and approval/PI meeting in SA			■	■	■									
Lab training in SA for sites 1 and 2				■	■									
EQA, enrolment and sample collection at site 1 and 2						■	■	■	■	■	■	■	■	■
Lab training in SA for sites 3 and 4												■		
EQA, enrolment and sample collection at site 3 and 4													■	■
Lab training in SA for sites 5 and 6														
EQA, enrolment and sample collection at site 5 and 6														
Lab training in SA for sites 7 and 8														
EQA, enrolment and sample collection at site 7 and 8														
Sample enrolment at site 9 (South Africa)														
Receiving of samples at RMPRU and processing. Three shippments per site									■	■	■	■	■	■
GBS serotyping, Quantitaitive analysis of swabs and antibody										■	■	■	■	■
Final data cleaning and analysis														

	2016											
	Jan	Feb	Mar	Apr	May	Jun	Jul	Aug	Sep	Oct	Nov	Dec
Protocol development/site selection/site application, with facilities description and draft budget												
Site IRB submission and approval/PI meeting in SA												
Lab training in SA for sites 1 and 2												
EQA, enrolment and sample collection at site 1 and 2												
Lab training in SA for sites 3 and 4												
EQA, enrolment and sample collection at site 3 and 4	■	■	■	■	■	■	■					
Lab training in SA for sites 5 and 6				■								
EQA, enrolment and sample collection at site 5 and 6					■	■	■	■	■	■	■	■
Lab training in SA for sites 7 and 8							■					
EQA, enrolment and sample collection at site 7 and 8												
Sample enrolment at site 9 (South Africa)												
Receiving of samples at RMPRU and processing. Three shippments per site	■	■	■	■	■	■	■	■	■	■	■	■
GBS serotyping, Quantitaitive analysis of swabs and antibody	■	■	■	■	■	■	■	■	■	■	■	■
Final data cleaning and analysis												

	2017												2018					
	Jan	Feb	Mar	Apr	May	Jun	Jul	Aug	Sep	Oct	Nov	Dec	Jan	Feb	Mar	Apr	May	Jun
Protocol development/site selection/site application, with facilities description and draft budget																		
Site IRB submission and approval/PI meeting in SA																		
Lab training in SA for sites 1 and 2																		
EQA, enrolment and sample collection at site 1 and 2																		
Lab training in SA for sites 3 and 4																		
EQA, enrolment and sample collection at site 3 and 4																		
Lab training in SA for sites 5 and 6																		
EQA, enrolment and sample collection at site 5 and 6	■																	
Lab training in SA for sites 7 and 8																		
EQA, enrolment and sample collection at site 7 and 8						■	■	■	■	■	■	■						
Sample enrolment at site 9 (South Africa)								■	■	■	■	■	■					
Receiving of samples at RMPRU and processing. Three shippments per site	■	■	■	■	■	■	■	■	■	■	■	■	■	■				
GBS serotyping, Quantitaitive analysis of swabs and antibody	■	■	■	■	■	■	■	■	■	■	■	■	■	■	■	■		
Final data cleaning and analysis																	■	■

12. References

1. Wang, H., et al., *Global, regional, and national levels of neonatal, infant, and under-5 mortality during 1990-2013: a systematic analysis for the Global Burden of Disease Study 2013.* Lancet, 2014.
2. Burman, L.G., et al., *Prevention of excess neonatal morbidity associated with group B streptococci by vaginal chlorhexidine disinfection during labour. The Swedish Chlorhexidine Study Group.* Lancet, 1992. **340**(8811): p. 65-9.
3. Madhi, S.A., et al., *Considerations for a phase-III trial to evaluate a group B Streptococcus polysaccharide-protein conjugate vaccine in pregnant women for the prevention of early- and late-onset invasive disease in young-infants.* Vaccine, 2013. **31 Suppl 4**: p. D52-7.
4. Schuchat, A., et al., *Risk factors and opportunities for prevention of early-onset neonatal sepsis: a multicenter case-control study.* Pediatrics, 2000. **105**(1 Pt 1): p. 21-6.
5. Darmstadt, G.L., et al., *Population-based incidence and etiology of community-acquired neonatal bacteremia in Mirzapur, Bangladesh: an observational study.* J Infect Dis, 2009. **200**(6): p. 906-15.
6. Schrag, S.J. and A. Schuchat, *Easing the burden: characterizing the disease burden of neonatal group B streptococcal disease to motivate prevention.* Clinical infectious diseases : an official publication of the Infectious Diseases Society of America, 2004. **38**(9): p. 1209-11.
7. Schrag, S.J., *Group B streptococcal vaccine for resource-poor countries.* Lancet, 2011. **378**(9785): p. 11-2.
8. Beal, S. and S. Dancer, *Antenatal prevention of neonatal group B streptococcal infection.* Reviews in Gynaecological and Perinatal Practice, 2006. **6**(3–4): p. 218-225.
9. Cutland, C.L., et al., *Chlorhexidine maternal-vaginal and neonate body wipes in sepsis and vertical transmission of pathogenic bacteria in South Africa: a randomised, controlled trial.* Lancet, 2009. **374**(9705): p. 1909-16.
10. Campbell, J.R., et al., *Group B streptococcal colonization and serotype-specific immunity in pregnant women at delivery.* Obstet Gynecol, 2000. **96**(4): p. 498-503.
11. Schrag, S.J. and J.R. Verani, *Intrapartum antibiotic prophylaxis for the prevention of perinatal group B streptococcal disease: experience in the United States and implications for a potential group B streptococcal vaccine.* Vaccine, 2013. **31 Suppl 4**: p. D20-6.
12. Kwatra G, C.M.C., Valencia C, Adrian P V, M Ip, Klugman KP, Madhi SA, Tam WH, Slobod KS., *MATERNAL COLONIZATION WITH GROUP B STREPTOCOCCUS:DO RATES VARY ACROSS REGIONS?* 8th World Congress of the World Society for Pediatric Infectious Diseases, 2013: p. Abstract no #291.
13. Edmond, K.M., et al., *Group B streptococcal disease in infants aged younger than 3 months: systematic review and meta-analysis.* Lancet, 2012. **379**(9815): p. 547-56.
14. Verani, J.R., L. McGee, and S.J. Schrag, *Prevention of perinatal group B streptococcal disease--revised guidelines from CDC, 2010.* MMWR Recomm Rep, 2010. **59**(RR-10): p. 1-36.
15. Konikkara, K.P., et al., *Evaluation of Culture, Antigen Detection and Polymerase Chain Reaction for Detection of Vaginal Colonization of Group B Streptococcus (GBS) in Pregnant Women.* J Clin Diagn Res, 2014. **8**(2): p. 47-9.
16. Patil KP, S.S., Nagmoti MB,. Swamy MK, *Group B Streptococci Colonization in Pregnant Women: Is Screening Necessary?* Journal of South Asian Federation of Obstetrics and Gynaecology, 2013(5(2):): p. 64-67.
17. Sharmila, V., et al., *Genital tract group B streptococcal colonization in pregnant women: a South Indian perspective.* Journal of infection in developing countries, 2011. **5**(8): p. 592-5.
18. Chan, G.J., et al., *Maternal and neonatal colonization in Bangladesh: prevalences, etiologies and risk factors.* J Perinatol, 2013. **33**(12): p. 971-6.
19. Dzowela T, K.O., Lgbigbia A, *Prevalence of group B Streptococcus colonization in antenatal women at the Queen Elizabeth Central Hospital Blantyre - a preliminary study.* Malawi Med J, 2005. **17**: p. 97-99.

20. Kwatra, G., et al., *Serotype-specific acquisition and loss of group B streptococcus recto-vaginal colonization in late pregnancy.* PLoS One, 2014. **9**(6): p. e98778.
21. Mavenyengwa, R.T., et al., *Group B Streptococcus colonization during pregnancy and maternal-fetal transmission in Zimbabwe.* Acta obstetricia et gynecologica Scandinavica, 2010. **89**(2): p. 250-5.
22. Mavenyengwa, R.T., et al., *Streptococcus agalactiae (group B streptococcus (GBS)) colonisation and persistence, in pregnancy; a comparison of two diverse communities (rural and urban).* The Central African journal of medicine, 2006. **52**(3-4): p. 38-43.
23. Madzivhandila, M., et al., *Serotype distribution and invasive potential of group B streptococcus isolates causing disease in infants and colonizing maternal-newborn dyads.* PloS one, 2011. **6**(3): p. e17861.
24. Baker, C.J., et al., *Maternal antibody at delivery protects neonates from early onset group B streptococcal disease.* J Infect Dis, 2014. **209**(5): p. 781-8.
25. Baker, C.J., M.S. Edwards, and D.L. Kasper, *Role of antibody to native type III polysaccharide of group B Streptococcus in infant infection.* Pediatrics, 1981. **68**(4): p. 544-9.
26. Baker, C.J. and D.L. Kasper, *Correlation of maternal antibody deficiency with susceptibility to neonatal group B streptococcal infection.* The New England journal of medicine, 1976. **294**(14): p. 753-6.
27. Lin, F.Y., et al., *Level of maternal antibody required to protect neonates against early-onset disease caused by group B Streptococcus type Ia: a multicenter, seroepidemiology study.* J Infect Dis, 2001. **184**(8): p. 1022-8.
28. Lin, F.Y., et al., *Level of maternal IgG anti-group B streptococcus type III antibody correlated with protection of neonates against early-onset disease caused by this pathogen.* J Infect Dis, 2004. **190**(5): p. 928-34.
29. Suara, R.O., et al., *Seroprevalence of antibodies to group B streptococcal polysaccharides in Gambian mothers and their newborns.* Journal of the National Medical Association, 1998. **90**(2): p. 109-14.
30. Poyart, C., et al., *Multiplex PCR assay for rapid and accurate capsular typing of group B streptococci.* J Clin Microbiol, 2007. **45**(6): p. 1985-8.
31. Bergh, K., et al., *Detection of group B streptococci (GBS) in vaginal swabs using real-time PCR with TaqMan probe hybridization.* Indian J Med Res, 2004. **119 Suppl**: p. 221-3.
32. Ke, D., et al., *Development of conventional and real-time PCR assays for the rapid detection of group B streptococci.* Clin Chem, 2000. **46**(3): p. 324-31.

13. Appendix 1

Sample collection and analyses overview

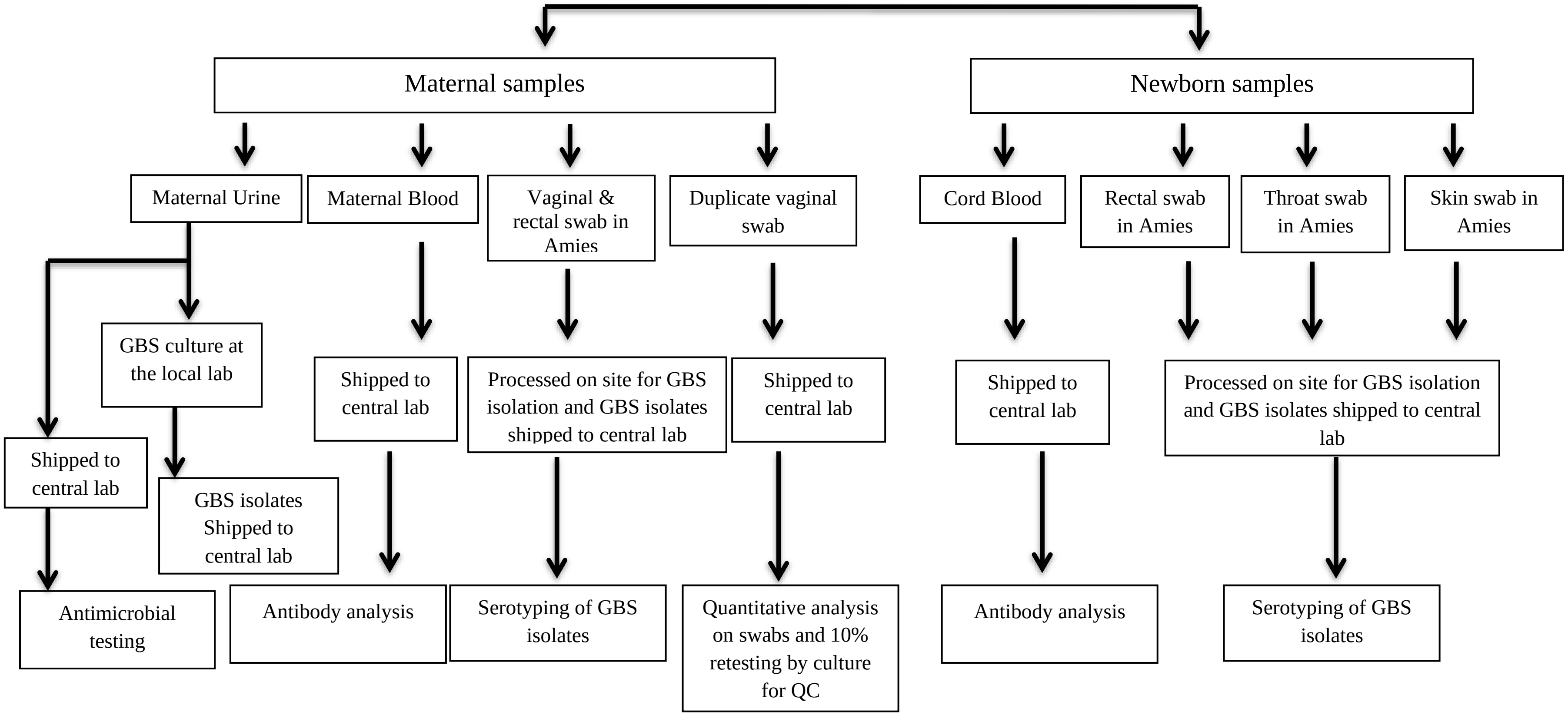

14. Appendix 2

External Quality assessment (EQA) Plan

EQA will be performed prior to the initiation of the collection of clinical samples. This will be done as follows:

- A materials pack will be shipped to each study site, and will include:
    - 20-30 EQA samples which consist of lyophilised mixtures of normal vaginal flora that are either positive or negative for GBS.
    - All laboratory material required to process these samples.
- Site laboratories will have two weeks to E-mail their results from the EQA samples.
- Approval of the site laboratory will be made within a week, depending on the results of the EQA. A pass mark of 90% will be considered acceptable.
- Sites which fail to achieve this pass mark will be re-evaluated as follows:
    - Problem samples will be investigated in order to determine the cause of the errors. For these purposes, site laboratories are encouraged to take photographs of the plates to assist us in resolving issues.
    - A site laboratory will be approved if the discordant results can be easily resolved telephonically.
    - Site laboratories with low scores that cannot be approved through minor procedural steps, will be visited by a representative from the central laboratory for further training and approval.

EQA will be extended throughout the trial as follows:

- All sites will collect duplicate vaginal swabs. One for immediate processing and the other for QC and qPCR purposes. The second swab will be stored on STGG media and shipped on dry ice to the central laboratory.
- 10% of maternal vaginal swabs will be re-processed by culture at the central laboratory.

- From previous EQA experience, 80% concordance is acceptable, due to the fact that the second swab may be different from the first, and that there may be some loss of viability due to storage and shipping.
- Discordant swabs will be analysed by qPCR for GBS. Discordant swabs with low copy number of GBS will be disregarded as these have a lower threshold for detection.
- Discordances if found will be investigated and communicated to the site laboratory.
